# Individual and combined effects of chemical and mechanical power on postoperative pulmonary complications: a secondary analysis of the REPEAT study

**DOI:** 10.1111/anae.16725

**Published:** 2025-08-19

**Authors:** Lukas M. Müller‐Wirtz, Thijs A. Lilien, William M. Patterson, Sascha Ott, Roland C. E. Francis, Marcelo Gama de Abreu, Ary Serpa Neto, Reinout A. Bem, David M. P. van Meenen, Marcus J. Schultz, for the REPEAT investigators, Sabrine N. T. Hemmes, Paolo Severgnini, Markus W. Hollmann, Jan M. Binnekade, Hermann Wrigge, Jaume Canet, Michael Hiesmayr, Werner Schmid, Edda Tschernko, Samir Jaber, Göran Hedenstierna, Christian Putensen, Paolo Pelosi, Agnes Marti, Alessandro Bacuzzi, Alexander Brodhun, Alexandre Molin, Alfred Merten, Ana Parera, Andrea Brunelli, Andrea Cortegiani, Andreas Güldner, Andreas W. Reske, Angelo Gratarola, Antonino Giarratano, Bea Bastin, Bjorn Heyse, Branka Mazul‐Sunko, Bruno Amantea, Bruno Barberis, Christopher Uhlig, Conrado Minguez Marín, Cristian Celentano, Daniela La Bella, David D’Antini, David Velghe, Demet Sulemanji, Edoardo De Robertis, Eric Hartmann, Francesca Montalto, Francesco Tropea, Gary H. Mills, Gilda Cinnella, Giorgio Della Rocca, Girolamo Caggianelli, Giulia Pellerano, Giuseppina Mollica, Guillermo Bugedo, Jan‐Paul Mulier, Jeroen Vandenbrande, Johann Geib, Jonathan Yaqub, Jorge Florez, Juan F. Mayoral, Juraj Sprung, Jurgen Van Limmen, Lieuwe D. J. Bos, Luc de Baerdemaeker, Luc Jamaer, Luigi Spagnolo, Lydia Strys, Manuel Granell Gil, Marcos F. Vidal Melo, Maria Carmen Unzueta, Maria Victoria Moral, Marion Ferner, Martin Weiss, Massimo Vanoni, Maximilian S. Schaefer, Mercè Prieto, Michele Grio, Peter Markus Spieth, Philipp Simon, Phoebe Bodger, Pilar Sierra, Rita Laufenberg‐Feldmann, Roberta Rusca, Rodolfo Proietti, Santi Maurizio Raineri, Santo Caroleo, Sergi Sabaté, Stefan De Hert, Stefano Pezzato, Tanja A. Treschan, Tatjana Goranovic, Thea Koch, Thomas Bluth, Thomas Kiss, Valter Perilli, Virginia Cegarra, Carlos Ferrando, Javier Belda, Marina Soro, Carmen Unzueta, Fernando Suarez‐Sipmann, Julián Librero, Alicia Llombart, Lucas Rovira, Manuel Granell, César Aldecoa, Oscar Diaz‐Cambronero, Jaume Balust, Ignacio Garutti, Rafael Gonzalez, Lucia Gallego, Santiago Garcia del Valle, Javier Redondo, David Pestaña, Aurelio Rodríguez, Javier García, Manuel de la Matta, Maite Ibáñez, Francisco Barrios, Samuel Hernández, Vicente Torres, Salvador Peiró, Natividad Pozo, Abigail Villena, Albert Carramiñana, Alberto Gallego‐Casilda, Alejandro Duca, Amalia Alcón, Amanda Miñana, Ana Asensio, Ana Colás, Ana Isabel Galve, Ana Izquierdo, Ana Jurado, Ana María Pérez, Ana Mugarra, Andrea Gutierrez, Ángeles De Miguel, Angels Lozano, Antonio Katime, Antonio Romero, Beatriz Garrigues, Begoña Ayas, Blanca Arocas, Carlos Delgado, Carmen Fernández, Carolina Romero, Clara Gallego, Cristina Garcés, Cristina Lisbona, Cristina Parrilla, Daniel López‐Herrera, Domingo González, Eduardo Llamazares, Elena Del Rio, Elena Lozano, Ernesto Pastor, Estefanía Chamorro, Estefanía Gracia, Ester Sánchez, Esther Romero, Fernando Díez, Ferran Serralta, Francisco Daviu, Francisco Sandín, Gerardo Aguilar, Gerardo Tusman, Gonzalo Azparren, Graciela Martínez‐Pallí, Guido Mazzinari, Inmaculada Benítez, Inmaculada Hernandéz, Inmaculada India, Irene León, Isabel Fuentes, Isabel Ruiz, Jaume Puig, Javie Ignacio Román, Jesús Acosta, Jesús Rico‐Feijoo, Jonathan Olmedo, Jose A. Carbonell, Jose M. Alonso, Jose María Pérez, Jose Miguel Marcos, Jose Navarro, Jose Valdivia, Juan Carrizo, Laura Piqueras, Laura Soriano, Laura Vaquero, Lisset Miguel, Lorena Muñoz, Lucia Valencia, Luis Olmedilla, Ma Justina Etulain, Manuel Tisner, María Barrio, María Dolores Alonso, María García, María J. Hernández, María José Alberola, María Parra, María Pilar Argente, María Vila, Mario De Fez, Marta Agilaga, Marta Gine, Mercedes Ayuso, Mercedes García, Natalia Bejarano, Natalia Peña, Nazario Ojeda, Nilda Martínez, Nuria García, Oto Padrón, Pablo García, Paola Valls, Patricia Cruz, Patricia Piñeiro, Pedro Charco, Rafael Anaya, Ramiro López, Rayco Rodríguez, Rocío Martínez, Roger Pujol, Rosa Dosdá, Rosa Lardies, Ruben Díaz, Rubén Villazala, Sara Zapatero, Sergio Cabrera, Sergio Sánchez, Silvia Martin, Suzana Diaz, Tania Franco, Tania Moreno, Tania Socorro, Vicente Gilabert, Victor Balandrón, Victoria Moral, Virgina Cegarra, Viviana Varón, Ilona Bobek, Cesare Gregoretti, John Laffey, Marc‐Joseph Licker, Klaus Markstaller, Idit Matot, Jan Paul Mulier, Rolf Rossaint, Jochen Schmitt, Mert Senturk, Fernando Abelha, Sühayla Abitağaoğlu, Marc Achilles, Afeez Adebesin, Ine Adriaensens, Charles Ahene, Fatima Akbar, Mohammed Al Harbi, Rita Al Khoury al Kallab, Xavier Albanel, Florence Aldenkortt, Rawan Abdullah Saleh Alfouzan, Reef Alruqaie, Fernando Altermatt, Bruno Luís de Castro Araujo, Genaro Arbesú, Hanna Artsi, Caterina Aurilio, Omer Hilmi Ayanoglu, Harris Baig, Yolanda Baird, Konstantin Balonov, Samantha Banks, Xiaodong Bao, Mélanie Baumgartner, Isabel Belda Tortosa, Alice Bergamaschi, Lars Bergmann, Luca Bigatello, Elena Biosca Pérez, Katja Birr, Elird Bojaxhi, Chiara Bonenti, Iwona Bonney, Elke M.E. Bos, Sara Bowman, Leandro Gobbo Braz, Elisa Brugnoni, Sorin J Brull, Iole Brunetti, Andrea Bruni, Shonie L. Buenvenida, Cornelius Johannes Busch, Giovanni Camerini, Beatrice Capatti, Javiera Carmona, Jaime Carungcong, Marta Carvalho, Anat Cattan, Carla Cavaleiro, Davide Chiumello, Stefano Ciardo, Mark Coburn, Umberto Colella, Victor Contreras, Pelin Corman Dincer, Elizabeth Cotter, Marcia Crovetto, William Darrah, Simon Davies, Enrique Del Cojo Peces, Ellise Delphin, John Diaper, Paulo do Nascimento Junior, Valerio Donatiello, Jing Dong, Maria do Socorro Dourado, Alexander Dullenkopf, Felix Ebner, Hamed Elgendy, Christoph Ellenberger, Dilek Erdoğan Arı, Thomas Ermert, Fadi Farah, Ana Fernandez‐Bustamante, Cristina Ferreira, Marco Fiore, Ana Fonte, Christina Fortià Palahí, Andrea Galimberti, Najia Garofano, Luca Gregorio Giaccari, Fernando Gilsanz, Felix Girrbach, Luca Gobbi, Marc Bernard Godfried, Nicolai Goettel, Peter A. Goldstein, Or Goren, Andrew Gorlin, Juan Graterol, Pierre Guyon, Kevin Haire, Philippe Harou, Antonia Helf, Gunther Hempel, Hernández Cádiz, María José, Ivan Huercio, Jasmina Ilievska, Lien Jakus, Vijay Jeganath, Yvonne Jelting, Minoa Jung, Barbara Kabon, Aalok Kacha, Maja Karaman Ilić, Arunthevaraja Karuppiah, Ayse Duygu Kavas, Gleicy Keli Barcelos, Todd A. Kellogg, Johann Kemper, Romain Kerbrat, Suraya Khodr, Peter Kienbaum, Bunyamin Kir, Selin Kivrak, Vlasta Klarić, Ceren Köksal, Ana Kowark, Peter Kranke, Bahar Kuvaki, Biljana Kuzmanovska, Mirko Lange, Marília Freitas de Lemos, Manuel López‐Baamonde, Antonio López‐Hernández, Mercedes Lopez‐Martinez, Stéphane Luise, Mark MacGregor, Danielle Magalhães, Julien Maillard, Patrizia Malerbi, Natesan Manimekalai, Michael Margarson, Archer K Martin, David P. Martin, Yvette N. Martin, Julia Martínez‐Ocon, Ignacio Martin‐Loeches, Emilio Maseda, Niamh McAuliffe, Travis J. McKenzie, Paulina Medina, Melanie Meersch, Angelika Menzen, Els Mertens, Bernd Meurer, Tanja Meyer‐Treschan, Changhong Miao, Camilla Micalizzi, Morena Milić, Norma Sueli Pinheiro Módolo, Pierre Moine, Patrick Mölders, Ana Montero‐Feijoo, Enrique Moret, Markus K. Muller, Zoe Murphy, Pramod Nalwaya, Filip Naumovski, Paolo Navalesi, Lais Helena Navarro e Lima, Višnja Nesek Adam, Claudia Neumann, Christopher Newell, Zoulfira Nisnevitch, Junaid Nizamuddin, Cecilia Novazzi, Michael O'Connor, Günther Oprea, Mukadder Orhan Sungur, Şule Özbilgin, Maria Caterina Pace, Marcos Pacheco, Balaji Packianathaswamy, Estefania Palma Gonzalez, Fotios Papaspyros, Sebastián Paredes, Maria Beatrice Passavanti, Juan Cristobal Pedemonte, Sanja Peremin, Christoph Philipsenburg, Daniela Pinho, Silvia Pinho, Linda M. Posthuma, Vincenzo Pota, Benedikt Preckel, Paolo Priani, Mohamed Aymen Rached, Aleksandar Radoeshki, Riccardo Ragazzi, Tamilselvan Rajamanickam, Arthi Rajamohan, Harish Ramakrishna, Desikan Rangarajan, Christian Reiterer, J. Ross Renew, Thomas Reynaud, Rhidian Rhys, Eva Rivas, Luisa Robitzky, Francesca Rubulotta, Humberto S. Machado, Catarina S. Nunes, Giovanni Sabbatini, Jon D Samuels, Josep Martí Sanahuja, Pasquale Sansone, Alice Santos, Mohamed Sayedalahl, Martin Scharffenberg, Eduardo Schiffer, Nadja Schliewe, Raoul Schorer, Roman Schumann, Gabriele Selmo, Mar Sendra, Kate Shaw, Mirjana Shosholcheva, Abdulrazak Sibai, Francesca Simonassi, Claudia Sinno, Nukhet Sivrikoz, Vasiliki Skandalou, Neil Smith, Maria Soares, Tania Socorro Artiles, Diogo Sousa Castro, Miguel Sousa, Savino Spadaro, Emmanouil Stamatakis, Luzius A. Steiner, Andrea Stevenazzi, Alejandro Suarez‐de‐la‐Rica, Mélanie Suppan, Robert Teichmann, José Maria Tena Guerrero, Bram Thiel, Raquel Tolós, Gulbin Tore Altun, Michelle Tucci, Zachary A. Turnbull, Žana Turudić, Matthias Unterberg, Yves Van Nieuwenhove, Julia Van Waesberghe, Bibiana Vitković, Luigi Vivona, Marcela Vizcaychipi, Carlo Alberto Volta, Anne Weber, Toby N. Weingarten, Jakob Wittenstein, Piet Wyffels, Julio Yagüe, David Yates, Ayşen Yavru, Lilach Zac, Jing Zhong

**Affiliations:** ^1^ Department of Anesthesiology Friedrich‐Alexander‐Universität Erlangen‐Nürnberg, Universitätsklinikum Erlangen Erlangen Germany; ^2^ Outcomes Research Consortium® Houston TX USA; ^3^ Department of Pediatric Intensive Care Amsterdam University Medical Center Amsterdam The Netherlands; ^4^ Department of Cardiac Anesthesiology and Intensive Care Medicine Deutsches Herzzentrum der Charité Berlin Germany; ^5^ Charité‐Universitätsmedizin Berlin, Corporate Member of Freie Universität Berlin, Humboldt‐Universität zu Berlin Berlin Germany; ^6^ Division of Cardiothoracic Critical Care; Division of Cardiothoracic Anesthesia Cleveland Clinic Cleveland OH USA; ^7^ Department of Anesthesiology Integrated Health Care Institute, Cleveland Clinic Cleveland OH USA; ^8^ Australian and New Zealand Intensive Care Research Centre (ANZIC‐RC), School of Public Health and Preventive Medicine Monash University Melbourne Australia; ^9^ Department of Intensive Care Austin Hospital Melbourne Australia; ^10^ Department of Anesthesiology Amsterdam University Medical Center Amsterdam The Netherlands; ^11^ Department of Intensive Care and Laboratory of Experimental Intensive Care and Anesthesiology Amsterdam University Medical Center Amsterdam The Netherlands; ^12^ Clinical Department of Cardiothoracic Vascular Surgery Anesthesia and Intensive Care Medicine Medical University Wien Vienna Austria

**Keywords:** anaesthesia, chemical power, mechanical power, oxygen, postoperative complications

## Abstract

**Introduction:**

Intra‐operative supplemental oxygen and mechanical ventilation expose the lungs to potentially injurious energy. This can be quantified as ‘chemical power’ and ‘mechanical power’, respectively. In this study, we sought to determine if intra‐operative chemical and mechanical power, individually and/or in combination, are associated with postoperative pulmonary complications.

**Methods:**

Using an individual patient data analysis of three randomised clinical trials of intra‐operative ventilation, we summarised intra‐operative chemical and mechanical power using time‐weighted averages. We evaluated the association between intra‐operative chemical and mechanical power and a collapsed composite of postoperative pulmonary complications using multivariable logistic regression to estimate the odds ratios related to the effect of 1 J.min^‐1^ increase in chemical or mechanical power with adjustment for demographic and intra‐operative characteristics. We also included an interaction term to assess for potential synergistic effects of chemical and mechanical power on postoperative pulmonary complications.

**Results:**

Of 3837 patients recruited to three individual trials, 2492 with full datasets were included in the analysis. Intra‐operative time‐weighted average (SD) chemical power was 10.2 (3.9) J.min^‐1^ and mechanical power was 10.5 (4.4) J.min^‐1^. An increase of 1 J.min^‐1^ in chemical power was associated with 8% higher odds of postoperative pulmonary complications (OR 1.08, 95%CI 1.05–1.10, p < 0.001), while the same increase in mechanical power raised odds by 5% (OR 1.05, 95%CI 1.02–1.08, p = 0.003). We did not find evidence of a significant interaction between chemical and mechanical power (p = 0.40), suggestive of an additive rather than synergistic effect on postoperative pulmonary complications.

**Discussion:**

Both chemical and mechanical power are independently associated with postoperative pulmonary complications. Further work is required to determine causality.

## Plain Language Summary

When people have surgery, they often need extra oxygen and a machine to help them breathe. But this can sometimes put stress on the lungs and cause problems afterward. In this study, doctors wanted to see if the amount of energy used by the oxygen and the breathing machine (called ‘chemical power’ and ‘mechanical power’) could be linked to lung problems after surgery. Doctors looked at data from three big studies involving patients who had surgery with breathing support. They measured how much chemical and mechanical power was used during surgery and averaged it over time. Then, they checked to see if people who had more of this power used during surgery were more likely to have breathing or lung problems afterward. They also checked if using both types of power together made things worse. Out of 3837 patients, 2492 had complete information and were included in the study. On average, the chemical power used was about 10.2 and the mechanical power was about 10.5 (measured in special units called Joules per minute). For every small increase in chemical power, the chance of lung problems after surgery went up by 8%. For the same increase in mechanical power, the chance went up by 5%. The two types of power didn't seem to make each other worse when used together. The study showed that both chemical and mechanical power during surgery can raise the risk of lung problems afterward. More research is needed to understand if one causes the other, or if something else is going on.

## Introduction

Intra‐operative ventilation transfers energy from the ventilator to lung tissue, which can be quantified as ‘mechanical power’ [1, 2]. Elevated intra‐operative mechanical power levels may harm the lungs and are associated with worse patient outcomes [3, 4]. Interventions that seek to reduce mechanical power are under consideration for patients who require intra‐operative ventilation [5]. However, less is known about the use of supplemental oxygen during intra‐operative ventilation, quantified as ‘chemical power’ [6]. The chemical power concept was introduced recently as a quantitative measure of the biochemical stress induced by hyperoxia and translates oxygen exposure (fraction of inspired oxygen, F_I_O_2_) into a power metric with units of joules per minute (J.min^‐1^). This involves estimation of pulmonary oxygen consumption; calculation of oxygen fraction converted into reactive oxygen species; and multiplication of the rate of reactive oxygen species production by the energy released per mole of superoxide formation [6].

Exposure to high chemical power is linked with postoperative pulmonary complications (PPCs); multiorgan injury; and mortality [7, 8]. The simplest way to reduce chemical power is to reduce F_I_O_2_ to the lowest safe level, including the use of recruitment manoeuvres and/or titration of positive end‐expiratory pressure (PEEP). Despite potential dangers of high intrapulmonary oxygen levels, high fractions of oxygen continue to be utilised during intra‐operative ventilation [9]. Clinical guidelines continue to recommend higher intra‐operative oxygen fractions [10, 11], justified by perceived safety margins for airway complications and limited evidence for a reduction in postoperative wound infections [12, 13]. Preclinical studies suggest that the combined effect of chemical and mechanical power may amplify the risk of pulmonary injury synergistically [14, 15], but the clinical relevance of this observation is uncertain.

Our aim was to evaluate how intra‐operative chemical and mechanical power, individually and in combination, influence the incidence of PPCs. We conducted a secondary analysis on the Re–Evaluation of the effects of high PEEP During General AnesThesia for surgery (REPEAT) database [16, 17]. This resource integrates individual patient data from three randomised clinical trials that evaluated the impact of intra‐operative ventilation with high PEEP on the incidence of PPCs. We hypothesised that both chemical and mechanical power are individually associated with PPCs, with a synergistic interaction.

## Methods

This is a secondary analysis of individual patient data from three randomised controlled trials that investigated the effect of low vs. high PEEP on PPCs: PROVHILO [18]; iPROVE [19]; and PROBESE [20]. The original trials were approved by a central institutional review board and all patients provided informed consent. Additional institutional review board approval or individual patient consent was required to access this pooled database. Our report adheres to the STROBE guideline.

For this secondary analysis, we did not include patients with missing data on the variables of interest (complete case analysis); duration of surgery < 2 h; and intra‐operative mechanical power > 30 J.min^‐1^. We extracted patient and surgery baseline characteristics: age; sex; height; weight; BMI; ASA physical status; ARISCAT score [21]; pre‐operative SpO_2_; respiratory infection; pre‐operative anaemia; history of heart failure; chronic obstructive pulmonary disease (COPD); active cancer; pre‐operative haemoglobin levels; surgical approach (open vs. laparoscopic); emergency procedure; duration of surgery; and surgical specialty. The following intra‐operative ventilatory variables were available and extracted in hourly intervals: tidal volume; respiratory rate; maximum airway pressure; PEEP; dynamic driving pressure; and F_I_O_2_. Patients were followed up for 7 days after surgery to detect PPCs in the original trials, as defined in online Supporting Information Table S1.

The two co‐primary exposures were defined as the time‐weighted average of intra‐operative chemical and mechanical power. Chemical power was calculated using the following equations [6]: Pulm_ROS_ = 1.7 × 10^‐5^ + ((F_I_O_2_ ‐ 0.21) × 1.63 × 10^‐4^) (mol.min^‐1^); chemical power = 141,000 × Pulm_ROS_ (J.min^‐1^), where Pulm_ROS_ is the local superoxide production in mol.min^‐1^. Dynamic driving pressure (ΔP) was used for mechanical power calculations, since plateau pressure was not available for all patients. Dynamic driving pressure was calculated with maximum airway pressure (P_max_) using the following equation: ΔP = P_max_ ‐ PEEP (cmH_2_O), where P_max_ and PEEP are expressed in cmH_2_O. Mechanical power was calculated using the following equation: mechanical power = 0.098 × tidal volume × respiratory rate × (P_max_ ‐ 0.5 × ΔP) (J.min^‐1^), where tidal volume in litres, respiratory rate in breaths.min^‐1^, P_max_ is maximum airway pressure in cmH_2_O and ΔP is dynamic driving pressure in cmH_2_O as described above.

The primary study endpoint was a composite of PPCs during the first seven postoperative days according to the definitions of the original trials presented in online Supporting Information Table S1. No formal power calculation was performed; instead, we used all available patients with complete data from the pooled dataset. For descriptive purposes only, the population was divided at the median of chemical power to create a ‘high chemical power group’ and a ‘low chemical power group’ and the absolute standardised difference was calculated to assess baseline balance. Inferential statistics were performed with continuous values of chemical and mechanical power.

Chemical and mechanical power were calculated hourly. We summarised intra‐operative chemical and mechanical power by calculating time‐weighted averages as the area under the chemical and mechanical power time curves divided by the number of hours of exposure for quantifying cumulative exposure for each patient. The association between intra‐operatively applied time‐weighted average chemical and mechanical power on a collapsed composite of PPCs was evaluated by multivariable logistic regression, estimating the odds ratios related to the effect of 1 J.min^‐1^ increase in chemical or mechanical power. Potential confounders were defined a priori and included as covariates in the multivariable model. To assess a potential interaction between chemical and mechanical power, we repeated the model with an interaction term for chemical and mechanical power.

We performed sensitivity analyses to explore our results. This included analyses restricted to patients with available plateau pressure; patients in whom F_I_O_2_ was likely set by default to 0.4, 0.5 or 0.8, i.e. without titration to the individual patient's oxygenation requirements; removal of exclusion criteria to include all patients in the database; and with adjustment for potential effects between individual trials. All analyses were performed based on an overall significance level of 0.05, using R (version 4.4.1, R Studio, Vienna, Austria).

## Results

**Figure 1:**
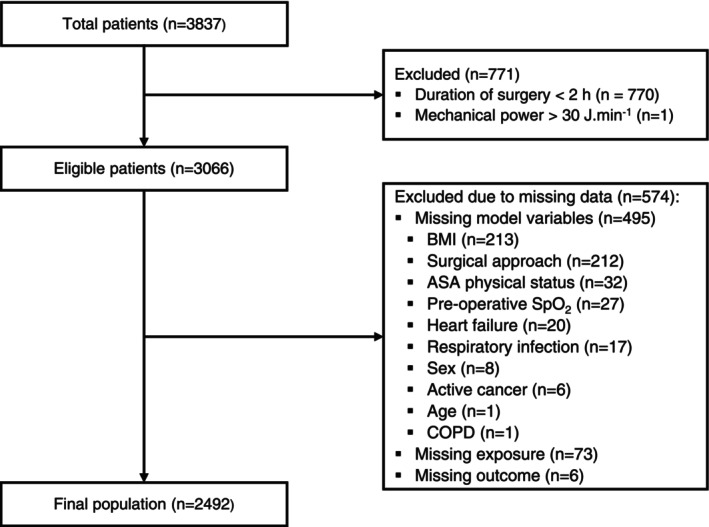
Study flow chart. COPD, chronic obstructive pulmonary disease; SpO_2_, peripheral oxygen saturation.

Of 3837 patients in the pooled database, 2492 were included in this analysis (Fig. 1). The main reasons for exclusion were duration of surgery < 2 h; missing BMI; and missing information on surgical approach. The mean (SD) patient age was 57 (15) y, 1300 (52%) were female and 1258 (50%) underwent colorectal or bariatric surgery (Table 1).

**Table 1:** Baseline characteristics for all included patients and for patients with below and above median chemical power. Values are mean (SD) or number (proportion).

	Overall n = 2492	Chemical power < 9 J.min^‐1^ n = 1532	Chemical power > 9 J.min^‐1^ n = 960	SMD
Age; y	57 (15)	55 (15)	62 (14)	0.496
Sex; female	1300 (52%)	882 (58%)	418 (44%)	0.283
Height; cm	170 (9.4)	170 (9.4)	170 (9.3)	0.099
Weight; kg	94 (31)	100 (30)	84 (29)	0.569
BMI	34 (10)	36 (10)	30 (9.6)	0.586
ASA physical status				0.077
1	144 (6%)	96 (6%)	48 (5%)	
2	1255 (50%)	783 (51%)	472 (49%)	
3	1069 (43%)	639 (42%)	430 (45%)	
4	24 (1%)	14 (1%)	10 (1%)	
ARISCAT score	38 (9.2)	40 (8.1)	36 (10)	0.468
Pre‐operative SpO_2_;%	97 (2.0)	97 (1.9)	97 (2.1)	0.148
Respiratory infection	113 (5%)	67 (4%)	46 (5%)	0.020
Pre‐operative anaemia	679 (27%)	337 (22%)	342 (36%)	0.304
Heart failure	201 (8%)	151 (10%)	50 (5%)	0.177
COPD	161 (6%)	110 (7%)	51 (5%)	0.077
Active cancer	1181 (47%)	511 (33%)	670 (70%)	0.783
Pre‐operative haemoglobin; g.dl^‐1^	13 (4.5)	14 (4.9)	13 (3.9)	0.111
Laparoscopic surgery	1138 (46%)	706 (46%)	432 (45%)	0.022
Emergency procedure	34 (1%)	30 (2%)	4 (0%)	0.143
Duration of surgery; min	220 (85)	210 (85)	230 (84)	0.176
Specific procedure				0.704
Abdominal/visceral	1153 (46%)	549 (36%)	604 (63%)	
Bariatric	685 (27%)	569 (37%)	116 (12%)	
Urologic	212 (9%)	132 (9%)	80 (8%)	
Gynaecologic	117 (5%)	71 (5%)	46 (5%)	
Vascular	45 (2%)	28 (2%)	17 (2%)	
Hernia	36 (1%)	27 (2%)	9 (1%)	
Other	244 (10%)	156 (10%)	88 (9%)	
Postoperative pulmonary complications	872 (35%)	444 (29%)	428 (45%)	

ARISCAT, Assess Respiratory Risk in Surgical Patients in Catalonia; COPD, chronic obstructive pulmonary disease; SMD, standardised mean difference; SpO_2_, peripheral oxygen saturation.

The time‐weighted averages of chemical and mechanical power were mean (SD) 10.2 (3.9) J.min^‐1^ and 10.5 (4.4) J.min^‐1^, respectively (Table 2). Patients who were administered higher chemical power were older; more often male; had a lower median BMI; and a lower median ARISCAT score (Table 1). The time‐weighted average mechanical power was similar in patients with low and high chemical power. Patients administered high chemical power tended to receive higher tidal volumes but lower respiratory rates (Table 2). Patients who experienced PPCs were more likely to have received higher intra‐operative chemical power (online Supporting Information Figure S1).

**Table 2:** Intra‐operative ventilation parameters for all included patients and for patients with below and above median chemical power. Values are mean (SD).

	Overall n = 2492	Chemical power < 9 J.min^‐1^ n = 1532	Chemical power > 9 J.min^‐1^ n = 960	SMD
Tidal volume; ml	460 (80)	460 (83)	470 (75)	0.208
Tidal volume; ml.kg PBW^‐1^	7.6 (0.8)	7.5 (0.7)	7.8 (0.8)	0.425
Respiratory rate; breaths.min^‐1^	14 (4)	15 (4)	14 (3)	0.224
Maximum airway pressure; cmH_2_O	25 (6)	24 (6)	25 (6)	0.110
PEEP; cmH_2_O	7.5 (4)	7.6 (5)	7.5 (4)	0.016
Dynamic driving pressure; cmH_2_O	17 (6)	17 (6)	18 (6)	0.124
F_I_O_2_; %	55 (17)	43 (4)	74 (10)	3.875
Chemical power; J.min^‐1^	10 (4)	7 (1)	15 (2)	3.875
Mechanical power; J.min^‐1^	10 (4)	10 (5)	10 (4)	0.011

F_I_O_2_, fraction of inspiratory oxygen; PBW, predicted bodyweight; PEEP, positive end‐expiratory pressure; SMD, standardised mean difference.

**Figure 2:**
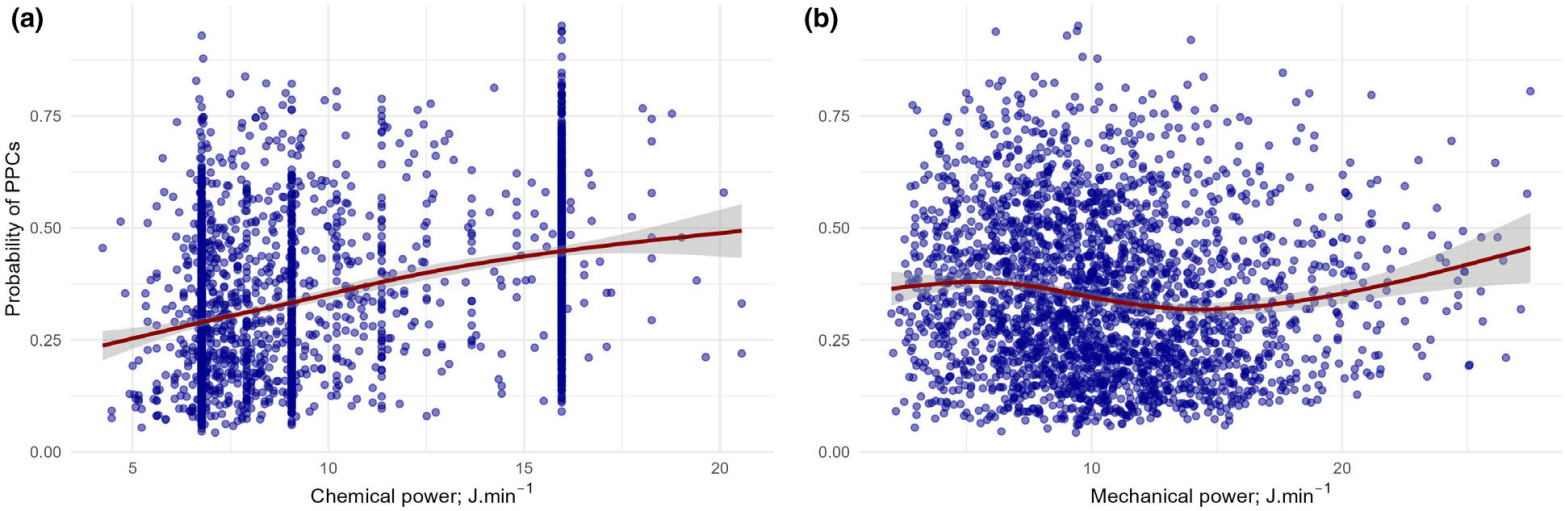
Probability of postoperative pulmonary complications (PPCs) over the ranges of (a) chemical and (b) mechanical power. Blue dots represent the probability of PPCs for each patient (n = 2492) based on the exposure to chemical or mechanical power, estimated using the primary confounder‐adjusted logistic regression model (Table 3). Smoothed curves with 95%CIs were added to highlight the trends in the average probability of postoperative pulmonary complications over the ranges of chemical and mechanical power (red lines with grey ranges).

**Figure 3:**
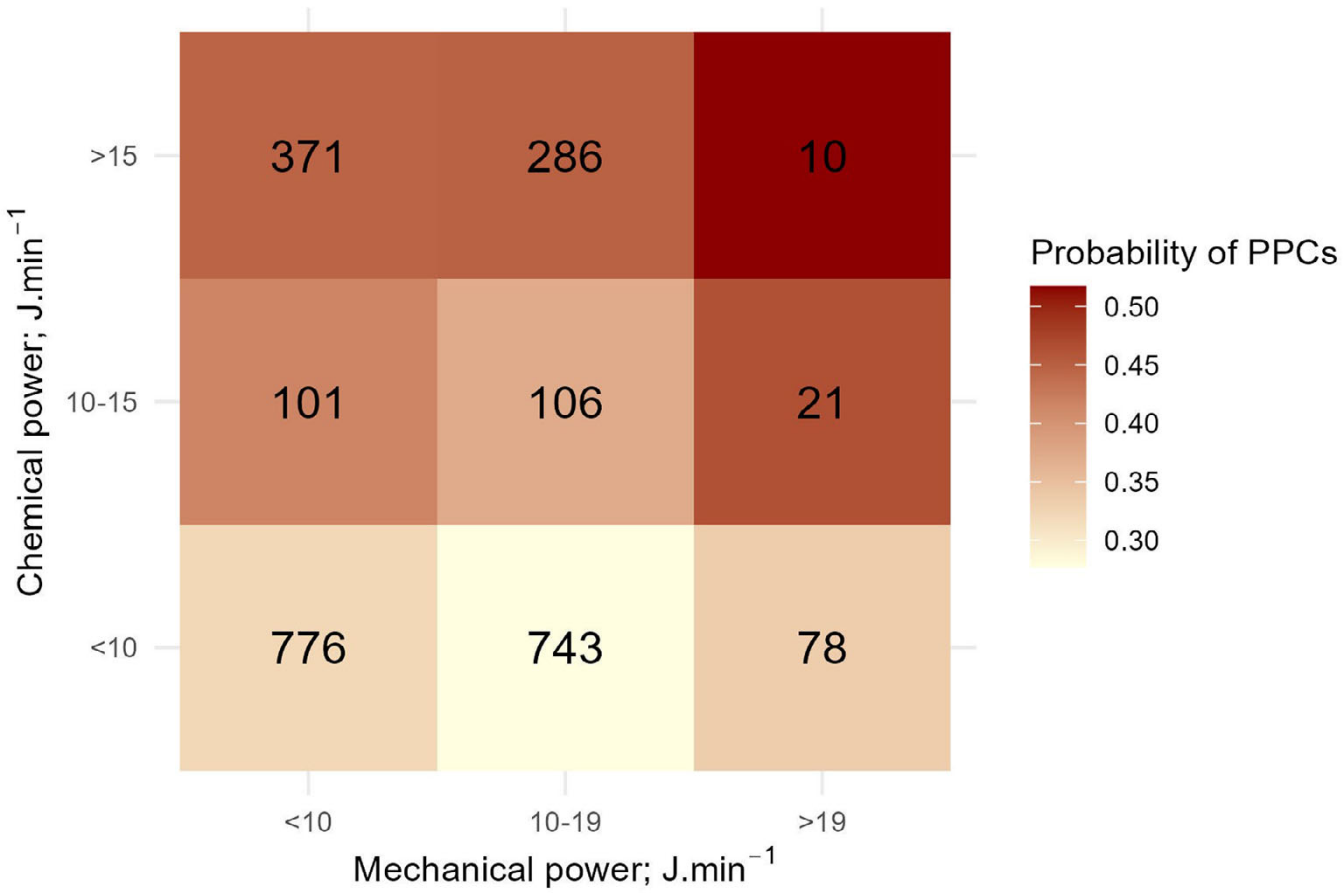
Probability of postoperative pulmonary complications (PPCs) associated with chemical and mechanical power. The population (n = 2492) was divided into 9 (3 × 3) bins of equally sized ranges of chemical and mechanical power (low, moderate, high). For each bin, the average probability of PPCs was estimated using the primary confounder‐adjusted logistic regression model (Table 3) and presented on a colour scale from light yellow to dark red as a 3 × 3 field heat map.

We observed that a 1 J.min^‐1^ increment in chemical power was associated with an 8% increased risk of PPCs (OR 1.08, 95%CI 1.05–1.10, p < 0.001). A 1 J.min^‐1^ increment in mechanical power was associated with a 5% increased risk of PPCs (OR 1.05, 95%CI 1.02–1.08, p < 0.003) (Table 3). The probability of PPCs increased linearly over the range of chemical power (Fig. 2a), whereas the probability of PPCs started to increase after 15 J.min^‐1^ of delivered mechanical power (Fig. 2b). Our model‐derived risk of PPCs suggests that chemical and mechanical power did not interact (p = 0.40, Fig. 3).

**Table 3:** Multivariable logistic regression model to assess the associations of chemical and mechanical power with postoperative pulmonary complications (n = 2492).

	Odds ratio	95%CI	p value
Chemical power; J.min^‐1^	1.08	1.05–1.10	< 0.001
Mechanical power; J.min^‐1^	1.05	1.02–1.08	0.003
Age; y	1.02	1.01–1.03	< 0.001
Sex; female	0.95	0.78–1.15	0.600
BMI	1.01	0.99–1.02	0.400
ASA physical status			
1	—	—	
2	1.94	1.22–3.18	0.006
3	2.84	1.77–4.72	< 0.001
4	2.24	0.83–6.09	0.110
Pre‐operative SpO_2_; %	0.92	0.87–0.96	< 0.001
Respiratory infection	1.54	1.01–2.33	0.042
Pre‐operative anaemia	1.05	0.86–1.30	0.600
Heart failure	1.36	0.98–1.88	0.064
COPD	1.12	0.79–1.59	0.500
Active cancer	0.92	0.72–1.16	0.500
Laparoscopic surgery	0.56	0.44–0.72	< 0.001
Emergency procedure	1.86	0.88–3.87	0.100
PEEP; cmH_2_O	0.97	0.94–0.99	0.008
Duration of surgery; min	1.01	1.00–1.01	< 0.001

COPD, chronic obstructive pulmonary disease; PEEP, positive end‐expiratory pressure; SpO_2_, peripheral oxygen saturation.

These findings were not altered in sensitivity analyses when we limited patients to those who had plateau pressures available (online Supporting Information Tables S2–S4 and Figures S2 and S3); and to patients in whom F_I_O_2_ was likely set by default to 0.4, 0.5 or 0.8 (i.e. without titration to the individual oxygenation requirements) (online Supporting Information Tables S5–7 and Figures S4 and S5). Similarly, findings were not altered when we extended our study population to include all patients in the database (online Supporting Information Tables S8–S10 and Figures S6 and S7) or included the individual trial as an independent variable in the regression analysis (online Supporting Information Table S11).

## Discussion

We found that chemical and mechanical power are individually associated with PPCs, but with additive rather than synergistic effects. This is important because both mechanical and chemical power are modifiable via optimisation of intra‐operative ventilator settings. Further work is required to determine if these observed associations are causal or predictive for postoperative lung injury. Given the high biological plausibility of the contribution of chemical and mechanical power to ventilator‐induced lung injury, we recommend titration of both parameters to aim for safe, rather than supranormal, respiratory physiological end points.

The evidence of pulmonary harm from high chemical power, or high F_I_O_2_, used for mechanical ventilation during surgery, is uncertain [7, 8]. Although older randomised trials found no effect of inspired oxygen on the incidence of PPCs [22, 23], more recent studies, with incorporation of lung‐protective ventilation, showed an increased risk of postoperative atelectasis and severe PPCs with 80% compared with 30% inspired oxygen [24, 25]. Regarding mechanical power, to date there are no randomised clinical trials that target mechanical power explicitly. Nevertheless, high mechanical power‐induced lung injury has been reported in animal studies [26, 27] and was repeatedly associated with postoperative lung injury [28, 29]. Our findings underline the need for large robust randomised clinical trials on intra‐operative chemical and mechanical power minimisation strategies.

Reduction of chemical power may seem straightforward in most patients by avoiding unnecessarily high F_I_O_2_ levels. However, patients prone to intra‐operative atelectasis, such as patients with obesity and patients undergoing laparoscopic procedures, may require recruitment manoeuvres and higher PEEP values to facilitate ventilation with low F_I_O_2_. In contrast, multiple interventions may reduce mechanical power, and it remains unclear which intervention works best [30, 31]. Recent studies underlined three essential concepts: lowering tidal volumes necessitates higher respiratory rates, potentially outweighing a tidal volume‐related reduction in mechanical power [32]; lowering respiratory rate effectively reduces mechanical power [33]; and permissive hypercapnia tolerated to facilitate lung‐protective ventilation protects the lungs [34, 35]. Therefore, permissive hypercapnic ventilation through low respiratory rates represents a potential strategy to reduce intra‐operative mechanical power, and warrants evaluation in future randomised trials.

Our study has several strengths and limitations. We developed our protocol a priori before conducting our analysis and this strengthens the scientific rigour of our report. A key strength is the use of robust prospectively collected clinical trial data to reduce the risk of undetected errors common in routine clinical documentation. The large sample size allowed us multiple adjustments for well‐established factors of pulmonary risk. Regarding limitations, the observational design of our study precludes definitive conclusions about causality. Although we adjusted for numerous potential confounders, the use of supplemental oxygen is likely confounded by underlying pulmonary conditions and individual responses to surgery and ventilation. Several hundred patients were not included due to missing covariate data. However, as missingness is likely random, we considered the risk of selection bias from a complete case analysis to be less significant than potential bias introduced by imputation. We have included a sensitivity analysis with inclusion of all patients in the database to explore this potential effect. The original studies used slightly different definitions of PPCs, introducing potential variability and bias to our results. Finally, our calculation of chemical power utilises F_I_O_2_ primarily; however, the underlying model also includes pulmonary oxygen consumption, which could be individualised at patient or group level in future studies.

In conclusion, both chemical and mechanical power are independently associated with PPCs. However, while chemical and mechanical power have an additive effect on the risk of PPCs, we did not observe a synergistic effect. Our findings contribute to the growing body of evidence emphasising the need for mechanical ventilation to be as ‘permissive’ as possible, minimising exposure to mechanical and chemical power.

## Supporting information


**Appendix S1.** REPEAT investigators.

**Table S1.** Definitions of postoperative pulmonary complications.
**Table S2.** Patient characteristics (including only patients with available plateau pressures).
**Table S3.** Intra‐operative ventilation parameters (including only patients with available plateau pressures).
**Table S4.** Multivariable regression model to assess the associations of chemical and mechanical power with postoperative pulmonary complications (including only patients with available plateau pressures).
**Table S5.** Patient characteristics (including only patients with F_I_O_2_ of 0.4, 0.5 or 0.8).
**Table S6.** Intra‐operative ventilation parameters (including only patients with F_I_O_2_ of 0.4, 0.5 or 0.8).
**Table S7.** Multivariable regression model to assess the associations of chemical and mechanical power with postoperative pulmonary complications (including only patients with F_I_O_2_ of 0.4, 0.5 or 0.8).
**Table S8.** Patient characteristics (including all patients).
**Table S9.** Intra‐operative ventilation parameters (including all patients).
**Table S10.** Multivariable regression model to assess the associations of chemical and mechanical power with postoperative pulmonary complications (including all patients).
**Table S11.** Multivariable regression model to assess the associations of chemical and mechanical power with postoperative pulmonary complications (adjustment of the primary analysis for trial effects).

**Figure S1.** Chemical power and the corresponding F_I_O_2_ in patients with and without postoperative pulmonary complications.
**Figure S2.** Probability of postoperative pulmonary complications over the ranges of chemical and mechanical power (including only patients with available plateau pressures).
**Figure S3.** Probability of postoperative pulmonary complications associated with chemical and mechanical power (including only patients with available plateau pressures).
**Figure S4.** Probability of postoperative pulmonary complications over the ranges of chemical and mechanical power (including only patients with F_I_O_2_ of 0.4, 0.5 or 0.8).
**Figure S5.** Probability of postoperative pulmonary complications associated with chemical and mechanical power (including only patients with F_I_O_2_ of 0.4, 0.5 or 0.8).
**Figure S6.** Probability of postoperative pulmonary complications over the ranges of chemical and mechanical power (including all patients).
**Figure S7.** Probability of postoperative pulmonary complications associated with chemical and mechanical power (including all patients).

Plain Language Summary.
