# Supplementary material for: Individual and combined effects of chemical and mechanical power on postoperative pulmonary complications: a secondary analysis of the REPEAT study
Source: Anaesthesia. 2025 Aug 19;80(12):1510–8. doi: 10.1111/anae.16725 (PMC12614417; doi:10.1111/anae.16725)
Supplement: Supplementary file 1 — Appendix S1. REPEAT investigators. [file ANAE-80-1510-s002.docx]

**Appendix S1** REPEAT investigators

**THE PROVHILO INVESTIGATORS**

*Steering and executive committees*

Sabrine NT Hemmes,^1^ Marcelo Gama de Abreu,^2^ Paolo Severgnini,^3^ Markus W Hollmann,^1^ Jan M Binnekade,^4^ Hermann Wrigge,^5^ Jaume Canet,^6^ Michael Hiesmayr,^7^ Werner Schmid,^7^ Edda Tschernko,^7^ Samir Jaber,^8^ Göran Hedenstierna,^9^ Christian Putensen,^10^ Paolo Pelosi,^11,12^ Marcus J Schultz^4,13,14^

**Amsterdam UMC, University of Amsterdam, Amsterdam, The Netherlands**

^1^Department of Anaesthesiology

^4^Department of Intensive Care

^14^Department of Intensive Care & Laboratory of Experimental Intensive Care and Anesthesiology (L·E·I·C·A)

**University Hospital Carl Gustav Carus, Technische Universität Dresden, Dresden, Germany**

^2^Pulmonary Engineering Group, Department of Anesthesiology and Intensive Care Medicine

**University of Insubria, Varese, Italy**

^3^Department of Biotechnologies and Sciences of Life

**University of Leipzig, Leipzig, Germany**

^5^Department of Anesthesiology and Intensive Care Medicine

**Hospital Universitari Germans Trias I Pujol, Barcelona, Spain**

^6^Department of Anesthesiology

**Medical University Vienna, Vienna, Austria**

^7^Division of Cardiac, Thoracic, and Vascular Anesthesia and Intensive Care

**Saint Eloi University Hospital, Montpellier, France**

^8^Department of Critical Care Medicine and Anesthesiology (SAR B)

**Uppsala University, Uppsala, Sweden**

^9^Department of Medical Sciences, Clinical Physiology

**University Hospital Bonn, Bonn, Germany**

^10^Dept. of Anesthesiology and Intensive Care Medicine

**^11^IRCCS San Martino Policlinico Hospital, Genoa, Italy**

**University of Genoa, Genoa, Italy**

^12^Department of Surgical Sciences and Integrated Diagnostics (DISC)

**Mahidol University, Bangkok, Thailand**

^13^Mahidol Oxford Tropical Medicine Research Unit (MORU), Faculty of Tropical Medicine

*Investigators*

| (**, indicates local principal investigators; names are listed in alphabetical order*) | |
| --- | --- |
| Agnes Marti | Hospital Universitari Germans Trias I Pujol, Spain |
| Alessandro Bacuzzi | University of Insubria - Azienda Ospedaliera Fondazione Macchi - Ospedale di Circolo, Italy |
| Alexander Brodhun | Johannes Gutenberg - Universität Mainz, Germany |
| Alexandre Molin* | Università degli Studi di Genova, IRCCS San Martino IST, Italy |
| Alfred Merten | Hospital Sant Pau, Spain |
| Ana Parera | Hospital Sant Pau, Spain |
| Andrea Brunelli* | Hospital Universitari Germans Trias I Pujol, Spain |
| Andrea Cortegiani | Università degli Studi di Palermo, Italy |
| Andreas Güldner | University Hospital Dresden, Technische Universität Dresden, Germany |
| Andreas W Reske | University of Leipzig, Germany |
| Angelo Gratarola | Università degli Studi di Genova, IRCCS San Martino IST, Italy |
| Antonino Giarratano* | Università degli Studi di Palermo, Italy |
| Bea Bastin | Düsseldorf University Hospital, Heinrich-Heine University Düsseldorf, Germany |
| Bjorn Heyse | Ghent University Hospital, Belgium |
| Branka Mazul-Sunko* | University Hospital Sveti Duh, Croatia |
| Bruno Amantea | University "Magna Graecia" of Catanzaro, Italy |
| Bruno Barberis | Azienda Sanitaria Locale TO3 - Ospedale di Rivoli, Italy |
| Christian Putensen* | University Hospital of Bonn Medical School, Germany |
| Christopher Uhlig | University Hospital Dresden, Technische Universität Dresden, Germany |
| Conrado Minguez Marín | Consorcio Hospital General Universitario Valencia, Spain |
| Cristian Celentano | Azienda Sanitaria Locale TO3 - Ospedale di Rivoli, Italy |
| Daniela La Bella | University of Foggia, Italy |
| David D’Antini | University of Foggia, Italy |
| David Velghe* | ZNA Middelheim, Belgium |
| Demet Sulemanji | Massachusetts General Hospital, USA |
| Edoardo De Robertis* | University of Napoli Federico II, Italy |
| Eric Hartmann | Johannes Gutenberg - Universität Mainz, Germany |
| Francesca Montalto | Università degli Studi di Palermo, Italy |
| Francesco Tropea | University "Magna Graecia" of Catanzaro, Italy |
| Gary H Mills* | Sheffield Teaching Hospitals, United Kingdom |
| Gilda Cinnella* | University of Foggia, Italy |
| Giorgio Della Rocca* | Università degli Studi di Udine, Italy |
| Girolamo Caggianelli | University of Foggia, Italy |
| Giulia Pellerano | Università degli Studi di Genova, IRCCS San Martino IST, Italy |
| Giuseppina Mollica | University of Foggia, Italy |
| Guillermo Bugedo* | Hospital Clínico de la Pontificia Universidad Católica de Chile, Chile |
| Hermann Wrigge* | University of Leipzig, Germany |
| Jan-Paul Mulier* | AZ St Jan, Belgium |
| Jeroen Vandenbrande | Virga Jesse Ziekenhuis, Belgium |
| Johann Geib | Düsseldorf University Hospital, Heinrich-Heine University Düsseldorf, Germany |
| Jonathan Yaqub | University Hospital Dresden, Technische Universität Dresden, Germany |
| Jorge Florez | Hospital Clínico de la Pontificia Universidad Católica de Chile, Chile |
| Juan F Mayoral | Fundación Puigvert, Spain |
| Juraj Sprung* | Mayo Clinic, USA |
| Jurgen Van Limmen | Ghent University Hospital, Belgium |
| Lieuwe DJ Bos | Academic Medical Center, University of Amsterdam, The Netherlands |
| Luc de Baerdemaeker | Ghent University Hospital, Belgium |
| Luc Jamaer* | Virga Jesse Ziekenhuis, Belgium |
| Luigi Spagnolo* | Azienda Sanitaria Locale TO3 - Ospedale di Rivoli, Italy |
| Lydia Strys | Johannes Gutenberg - Universität Mainz, Germany |
| Manuel Granell Gil* | Consorcio Hospital General Universitario Valencia, Spain |
| Marcelo Gama de Abreu* | University Hospital Dresden, Technische Universität Dresden, Germany |
| Marcos F Vidal Melo* | Massachusetts General Hospital, USA |
| Marcus J Schultz* | Academic Medical Center, University of Amsterdam, The Netherlands |
| Maria Carmen Unzueta* | Hospital Sant Pau, Spain |
| Maria Victoria Moral | Hospital Sant Pau, Spain |
| Marion Ferner | Johannes Gutenberg - Universität Mainz, Germany |
| Markus W Hollmann | Academic Medical Center, University of Amsterdam, The Netherlands |
| Martin Weiss | Düsseldorf University Hospital, Heinrich-Heine University Düsseldorf, Germany |
| Massimo Vanoni | University of Insubria - Azienda Ospedaliera Fondazione Macchi - Ospedale di Circolo, Italy |
| Maximilian S Schaefer | Düsseldorf University Hospital, Heinrich-Heine University Düsseldorf, Germany |
| Mercè Prieto | Fundación Puigvert, Spain |
| Michele Grio | Azienda Sanitaria Locale TO3 - Ospedale di Rivoli, Italy |
| Paolo Severgnini* | University of Insubria - Azienda Ospedaliera Fondazione Macchi - Ospedale di Circolo, Italy |
| Peter Markus Spieth | University Hospital Dresden, Technische Universität Dresden, Germany |
| Philipp Simon | University of Leipzig, Germany |
| Phoebe Bodger* | Barts Health NHS Trust, United Kingdom |
| Pilar Sierra | Fundación Puigvert, Spain |
| Rita Laufenberg-Feldmann* | Johannes Gutenberg - Universität Mainz, Germany |
| Roberta Rusca | Università degli Studi di Genova, IRCCS San Martino IST, Italy |
| Rodolfo Proietti* | Università degli Studi di Roma Cattolica, Italy |
| Sabrine NT Hemmes | Academic Medical Center, University of Amsterdam, The Netherlands |
| Santi Maurizio Raineri | Università degli Studi di Palermo, Italy |
| Santo Caroleo* | University "Magna Graecia" of Catanzaro, Italy |
| Sergi Sabaté* | Fundación Puigvert, Spain |
| Stefan De Hert* | Ghent University Hospital, Belgium |
| Stefano Pezzato | Università degli Studi di Genova, IRCCS San Martino IST, Italy |
| Tanja A Treschan* | Düsseldorf University Hospital, Heinrich-Heine University Düsseldorf, Germany |
| Tatjana Goranovic | University Hospital Sveti Duh, Croatia |
| Thea Koch | University Hospital Dresden, Technische Universität Dresden, Germany |
| Thomas Bluth | University Hospital Dresden, Technische Universität Dresden, Germany |
| Thomas Kiss | University Hospital Dresden, Technische Universität Dresden, Germany |
| Valter Perilli | Università degli Studi di Roma Cattolica, Italy |
| Virginia Cegarra | Hospital Sant Pau, Spain |
| Werner Schmid* | Medical University Viena, Viena |

*Information and funding*

The PROVHILO trial is a collaboration of the Protective Ventilation Network (PROVENet). The trial was funded by the European Society of Anaesthesiology (ESA) and the Academical Medical Center (AMC, Amsterdam, The Netherlands).

**THE iPROVE INVESTIGATORS**

*Steering and executive committees*

Carlos Ferrando,^1,2^ Javier Belda,^3^ Marina Soro,^4^ Jaume Canet,^5^ Carmen Unzueta,^6^ Fernando Suarez-Sipmann,^7^ Julián Librero,^8^ Alicia Llombart,^9^ Lucas Rovira,^10^ Manuel Granell,^10^ César Aldecoa,^11^ Oscar Diaz-Cambronero,^12^ Jaume Balust,^13^ Ignacio Garutti,^14^ Rafael Gonzalez,^15^ Lucia Gallego,^16^ Santiago Garcia del Valle,^17^ Javier Redondo,^18^ David Pestaña,^19^ Aurelio Rodríguez,^20^ Javier García,^21^ Manuel de la Matta,^22^ Maite Ibáñez,^23^ Francisco Barrios,^24^ Samuel Hernández,^25^ Vicente Torres,^26^ Salvador Peiró,^27^ Natividad Pozo^28^

**Hospital Clinic de Barcelona, Barcelona, Spain**

^1^Department of Anesthesiology and Critical Care

**^2^CIBER of Respiratory Disease, Instituto de Salud Carlos III, Madrid, Spain**

**Medicine Faculty, Universidad de Valencia, Valencia, Spain**

^3^Department of Surgery

**Hospital Clínico Universitario de Valencia, Valencia, Spain**

^4^Department of Anesthesiology and Critical Care

**Hospital Universitario Germans Tries i Pujol, Barcelona, Spain**

^5^Department of Anesthesiology and Critical Care

**Hospital Universitario San Pau, Barcelona, Spain**

^6^Department of Anesthesiology and Critical Care

**Hospital Universitario La Princesa, Madrid, Spain**

^7^Department of Anesthesiology and Critical Care

**Red de Investigación en Servicios de Salud en Enfermedades Crónicas (REDISSEC), Pamplona, Spain**

^8^Navarrabiomed-Fundación Miguel Servet

**Corachan Hospital, Barcelona, Spain**

^9^Department of Pharmacology

**Hospital General Universitario de Valencia, Valencia, Spain**

^10^Department of Anesthesiology and Critical Care

**Hospital Universitario Río Hortega, Valladolid, Spain**

^11^Department of Anesthesiology and Critical Care

**Hospital Universitario y Politécnico La Fe, Valencia, Spain**

^12^Department of Anesthesiology and Critical Care

^27^IISLAFE Clinical Research Institute

**Hospital Clínic i Provincial Universitario de Barcelona, Barcelona, Spain**

^13^Department of Anesthesiology and Critical Care

**Hospital General Universitario Gregorio Marañón, Madrid, Spain**

^14^Department of Anesthesiology and Critical Care

**Hospital de León, León, Spain**

^15^Department of Anesthesiology and Critical Care

**Hospital Universitario Miguel Servet de Zaragoza, Zaragoza, Spain**

^16^Department of Anesthesiology and Critical Care

**Hospital Fundación de Alcorcón, Alcorcón, Spain**

^17^Department of Anesthesiology and Critical Care

**Hospital General Universitario de Ciudad Real, Ciudad Real, Spain**

^18^Department of Anesthesiology and Critical Care

**Hospital Universitario Ramón y Cajal, Madrid, Spain**

^19^Department of Anesthesiology and Critical Care

**Hospital de Gran Canaria, Dr. Negrín, Gran Canaria, Spain**

^20^Department of Anesthesiology and Critical Care

**Hospital Universitario Puerta de Hierro de Majadahonda, Madrid, Spain**

^21^Department of Anesthesiology and Critical Care

**Hospital Universitario Virgen del Rocio de Sevilla, Sevilla, Spain**

^22^Department of Anesthesiology and Critical Care

**Hospital de la Marina Baixa de la Vila Joiosa, Alicante, Spain**

^23^Department of Anesthesiology

**Hospital Principe de Asturias, Madrid, Spain**

^24^Department of Anesthesiology and Critical Care

**Hospital NS de Candelaria, Santa Cruz de Tenerife, Spain**

^25^Department of Anesthesiology

**Hospital Son Espases de Mallorca, Mallorca, Spain**

^26^Department of Anesthesiology and Critical Care

**^28^INCLIVA, Biomedical Research Institute, Valencia, Spain**

*Investigators*

| (*names are listed in alphabetical order*) | |
| --- | --- |
| Abigail Villena | Hospital Clínico Universitario de Valencia, Valencia, Spain |
| Albert Carramiñana | Hospital Universitario Germans Tries i Pujol, Badalona, Spain |
| Alberto Gallego-Casilda | Hospital Universitario Miguel Servet de Zaragoza, Zaragoza, Spain |
| Alejandro Duca | Hospital Clínico Universitario de Valencia, Valencia, Spain |
| Amalia Alcón | Hospital Clínic i Provincial Universitario de Barcelona, Barcelona, Spain |
| Amanda Miñana | Hospital Clínico Universitario de Valencia, Valencia, Spain |
| Ana Asensio | Hospital Universitario Miguel Servet de Zaragoza, Zaragoza, Spain |
| Ana Colás | Hospital Universitario Miguel Servet de Zaragoza, Zaragoza, Spain |
| Ana Isabel Galve | Hospital General Universitario Gregorio Marañón, Madrid, Spain |
| Ana Izquierdo | Hospital Clínico Universitario de Valencia, Valencia, Spain |
| Ana Jurado | Hospital Clínico Universitario de Valencia, Valencia, Spain |
| Ana María Pérez | Hospital de León, León, Spain |
| Ana Mugarra | Hospital Clínico Universitario de Valencia, Valencia, Spain |
| Ana Parera | Hospital Universitario San Pau, Barcelona, Spain |
| Andrea Brunelli | Hospital Universitario Germans Tries i Pujol, Badalona, Spain |
| Andrea Gutierrez | Hospital Clínico Universitario de Valencia, Valencia, Spain |
| Ángeles De Miguel | Hospital General Universitario Gregorio Marañón, Madrid, Spain |
| Angels Lozano | Hospital Clínico Universitario de Valencia, Valencia, Spain |
| Antonio Katime | Hospital Clínico Universitario de Valencia, Valencia, Spain |
| Antonio Romero | Hospital Universitario Puerta de Hierro de Majadahonda, Madrid, Spain |
| Beatriz Garrigues | Hospital Clínico Universitario de Valencia, Valencia, Spain |
| Begoña Ayas | Hospital Universitario y Politécnico La Fe, Valencia, Spain |
| Blanca Arocas | Hospital Clínico Universitario de Valencia, Valencia, Spain |
| Carlos Delgado | Hospital Clínico Universitario de Valencia, Valencia, Spain |
| Carmen Fernández | Hospital General Universitario Gregorio Marañón, Madrid, Spain |
| Carolina Romero | Hospital Clínico Universitario de Valencia, Valencia, Spain |
| Clara Gallego | Hospital Universitario Ramón y Cajal, Madrid, Spain |
| Cristina Garcés | Hospital Universitario Miguel Servet de Zaragoza, Zaragoza, Spain |
| Cristina Lisbona | Hospital General Universitario Gregorio Marañón, Madrid, Spain |
| Cristina Parrilla | Hospital Clínico Universitario de Valencia, Valencia, Spain |
| Daniel López-Herrera | Hospital Universitario Virgen del Rocio de Sevilla, Sevilla, Spain |
| Domingo González | Hospital Universitario Virgen del Rocio de Sevilla, Sevilla, Spain |
| Eduardo Llamazares | Hospital General Universitario Gregorio Marañón, Madrid, Spain |
| Elena Del Rio | Hospital Clínico Universitario de Valencia, Valencia, Spain |
| Elena Lozano | Hospital de la Marina Baixa de la Vila Joiosa, Alicante, Spain |
| Ernesto Pastor | Hospital Clínico Universitario de Valencia, Valencia, Spain |
| Estefanía Chamorro | Hospital General Universitario Gregorio Marañón, Madrid, Spain |
| Estefanía Gracia | Hospital Clínico Universitario de Valencia, Valencia, Spain |
| Ester Sánchez | Hospital de la Marina Baixa de la Vila Joiosa, Alicante, Spain |
| Esther Romero | Hospital Clínico Universitario de Valencia, Valencia, Spain |
| Fernando Díez | Hospital de León, León, Spain |
| Ferran Serralta | Hospital Clínico Universitario de Valencia, Valencia, Spain |
| Francisco Daviu | Hospital General Universitario Gregorio Marañón, Madrid, Spain |
| Francisco Sandín | Hospital Universitario Miguel Servet de Zaragoza, Zaragoza, Spain |
| Gerardo Aguilar | Hospital Clínico Universitario de Valencia, Valencia, Spain |
| Gerardo Tusman | Hospital Privado de Comunidad, Mar del Plata, Buenos Aires, Argentina |
| Gonzalo Azparren | Hospital Universitario San Pau, Barcelona, Spain |
| Graciela Martínez-Pallí | Hospital Clínic i Provincial Universitario de Barcelona, Barcelona, Spain |
| Guido Mazzinari | Hospital de Manises, Valencia, Spain |
| Inmaculada Benítez | Hospital Universitario Virgen del Rocio de Sevilla, Sevilla, Spain |
| Inmaculada Hernandéz | Hospital General Universitario Gregorio Marañón, Madrid, Spain |
| Inmaculada India | Hospital Universitario San Pau, Barcelona, Spain |
| Irene León | Hospital Clínic i Provincial Universitario de Barcelona, Barcelona, Spain |
| Isabel Fuentes | Hospital Clínico Universitario de Valencia, Valencia, Spain |
| Isabel Ruiz | Hospital Universitario Ramón y Cajal, Madrid, Spain |
| Jaume Puig | Hospital Clínico Universitario de Valencia, Valencia, Spain |
| Javie Ignacio Román | Hospital Son Espases de Mallorca, Mallorca, Spain |
| Jesús Acosta | Hospital Universitario Virgen del Rocio de Sevilla, Sevilla, Spain |
| Jesús Rico-Feijoo | Hospital Universitario Río Hortega, Valladolid, Spain |
| Jonathan Olmedo | Hospital General Universitario Gregorio Marañón, Madrid, Spain |
| Jose A. Carbonell | Hospital Clínico Universitario de Valencia, Valencia, Spain |
| Jose M. Alonso | Hospital Clínico Universitario de Valencia, Valencia, Spain |
| Jose María Pérez | Hospital General Universitario Gregorio Marañón, Madrid, Spain |
| Jose Miguel Marcos | Hospital de León, León, Spain |
| Jose Navarro | Hospital Clínico Universitario de Valencia, Valencia, Spain |
| Jose Valdivia | Hospital de la Marina Baixa de la Vila Joiosa, Alicante, Spain |
| Juan Carrizo | Hospital Clínico Universitario de Valencia, Valencia, Spain |
| Laura Piqueras | Hospital Clínico Universitario de Valencia, Valencia, Spain |
| Laura Soriano | Hospital General Universitario Gregorio Marañón, Madrid, Spain |
| Laura Vaquero | Hospital Universitario Río Hortega, Valladolid, Spain |
| Lisset Miguel | Hospital Universitario Ramón y Cajal, Madrid, Spain |
| Lorena Muñoz | Hospital General Universitario de Valencia, Valencia, Spain |
| Lucia Valencia | Hospital de Gran Canaria, Dr. Negrín, Gran Canaria, Spain |
| Luis Olmedilla | Hospital General Universitario Gregorio Marañón, Madrid, Spain |
| M^a^ Justina Etulain | Hospital General Universitario Gregorio Marañón, Madrid, Spain |
| Manuel Tisner | Hospital General Universitario Gregorio Marañón, Madrid, Spain |
| María Barrio | Hospital General Universitario Gregorio Marañón, Madrid, Spain |
| María Dolores Alonso | Hospital General Universitario de Valencia, Valencia, Spain |
| María García | Hospital Universitario Río Hortega, Valladolid, Spain |
| María J. Hernández | Hospital General Universitario de Valencia, Valencia, Spain |
| María José Alberola | Hospital Universitario y Politécnico La Fe, Valencia, Spain |
| María Parra | Hospital Clínico Universitario de Valencia, Valencia, Spain |
| María Pilar Argente | Hospital Universitario y Politécnico La Fe, Valencia, Spain |
| María Vila | Hospital Universitario y Politécnico La Fe, Valencia, Spain |
| Mario De Fez | Hospital Clínico Universitario de Valencia, Valencia, Spain |
| Marta Agilaga | Hospital Universitario San Pau, Barcelona, Spain |
| Marta Gine | Hospital Universitario San Pau, Barcelona, Spain |
| Mercedes Ayuso | Hospital Principe de Asturias, Madrid, Spain |
| Mercedes García | Hospital Universitario San Pau, Barcelona, Spain |
| Natalia Bejarano | Hospital General Universitario de Ciudad Real, Ciudad Real, Spain |
| Natalia Peña | Hospital Universitario Miguel Servet de Zaragoza, Zaragoza, Spain |
| Nazario Ojeda | Hospital de Gran Canaria, Dr. Negrín, Gran Canaria, Spain |
| Nilda Martínez | Hospital Universitario Ramón y Cajal, Madrid, Spain |
| Nuria García | Hospital Universitario y Politécnico La Fe, Valencia, Spain |
| Oto Padrón | Hospital de Gran Canaria, Dr. Negrín, Gran Canaria, Spain |
| Pablo García | Hospital Fundación de Alcorcón, Alcorcón, Spain |
| Paola Valls | Hospital Clínico Universitario de Valencia, Valencia, Spain |
| Patricia Cruz | Hospital General Universitario Gregorio Marañón, Madrid, Spain |
| Patricia Piñeiro | Hospital General Universitario Gregorio Marañón, Madrid, Spain |
| Pedro Charco | Hospital Clínico Universitario de Valencia, Valencia, Spain |
| Rafael Anaya | Hospital Universitario San Pau, Barcelona, Spain |
| Ramiro López | Hospital General Universitario Gregorio Marañón, Madrid, Spain |
| Rayco Rodríguez | Hospital de Gran Canaria, Dr. Negrín, Gran Canaria, Spain |
| Rocío Martínez | Hospital General Universitario Gregorio Marañón, Madrid, Spain |
| Roger Pujol | Hospital Clínic i Provincial Universitario de Barcelona, Barcelona, Spain |
| Rosa Dosdá | Hospital Clínico Universitario de Valencia, Valencia, Spain |
| Rosa Lardies | Hospital Clínic i Provincial Universitario de Barcelona, Barcelona, Spain |
| Ruben Díaz | Hospital Universitario San Pau, Barcelona, Spain |
| Rubén Villazala | Hospital General Universitario de Ciudad Real, Ciudad Real, Spain |
| Sara Zapatero | Hospital General Universitario Gregorio Marañón, Madrid, Spain |
| Sergio Cabrera | Hospital de Gran Canaria, Dr. Negrín, Gran Canaria, Spain |
| Sergio Sánchez | Hospital General Universitario de Ciudad Real, Ciudad Real, Spain |
| Silvia Martin | Hospital Universitario Río Hortega, Valladolid, Spain |
| Suzana Diaz | Hospital General Universitario Gregorio Marañón, Madrid, Spain |
| Tania Franco | Hospital Universitario Ramón y Cajal, Madrid, Spain |
| Tania Moreno | Hospital Clínico Universitario de Valencia, Valencia, Spain |
| Tania Socorro | Hospital Clínico Universitario de Valencia, Valencia, Spain |
| Vicente Gilabert | Hospital de la Marina Baixa de la Vila Joiosa, Alicante, Spain |
| Victor Balandrón | Hospital General Universitario de Ciudad Real, Ciudad Real, Spain |
| Victoria Moral | Hospital Universitario San Pau, Barcelona, Spain |
| Virgina Cegarra | Hospital Universitario San Pau, Barcelona, Spain |
| Viviana Varón | Hospital Fundación de Alcorcón, Alcorcón, Spain |

*Information and funding*

The iPROVE trial wants to thank all staff who participated, including researchers, nurses, emergency medical technicians, and anyone who has made possible the successful completion of the project. We also thank the technical support provided by the Biomedical Research Foundation Institute. This trial was funded by the Instituto de Salud Carlos III of the Spanish Ministry of Economy and Competitiveness (grant PI14/00829, co-financed by the European Regional Development Fund), and received the support of the Grants Programme of the European Society of Anaesthesiology.

**THE PROBESE INVESTIGATORS**

*Writing, steering and executive committees*

Thomas Bluth,^1^ Ary Serpa Neto,^2,3,4^ Ilona Bobek,^5^ Jaume Canet,^6^ Gilda Cinnella,^7^ Luc de Baerdemaeker,^8^ Cesare Gregoretti,^9^ Göran Hedenstierna,^10^ Sabrine NT Hemmes,^11^ Michael Hiesmayr,^12^ Markus W Hollmann,^11^ Samir Jaber,^13^ John Laffey,^14^ Marc-Joseph Licker,^15^ Klaus Markstaller,^16^ Idit Matot,^17^ Gary H Mills,^18^ Jan Paul Mulier,^19^ Christian Putensen,^20^ Rolf Rossaint,^21^ Jochen Schmitt,^22^ Mert Senturk,^23^ Paolo Severgnini,^24^ Juraj Sprung,^25^ Marcos F Vidal Melo,^26^ Hermann Wrigge,^27,28^ Marcus J Schultz,^3,29^ Paolo Pelosi,^30,31^ Marcelo Gama de Abreu^1^

**University Hospital Carl Gustav Carus, Technische Universität Dresden, Dresden, Germany**

^1^Pulmonary Engineering Group, Department of Anesthesiology and Intensive Care Medicine

^22^Center for Evidence-based Healthcare

**Hospital Israelita Albert Einstein, São Paulo, Brazil**

^2^Department of Critical Care Medicine

**Amsterdam UMC, University of Amsterdam, Amsterdam, The Netherlands** ^3^Department of Intensive Care & Laboratory of Experimental Intensive Care and Anesthesiology (L·E·I·C·A)

^11^Department of Anaesthesiology

**Instituto do Coração, Hospital das Clinicas HCFMUSP, Faculdade de Medicina, Universidade de Sao Paulo, Sao Paulo, Brazil**

^4^Cardio-Pulmonary Department, Pulmonary Division

**Semmelweis Egyetem, Budapest, Hungary**

^5^Aneszteziológiai és Intenzív Terápiás Klinika

**Hospital Universitari Germans Trias I Pujol, Barcelona, Spain**

^6^Department of Anesthesiology

**Unviersity of Foggia, Foggia, Italy**

^7^Department of Anesthesiology and Intensive Care Medicine

**Ghent University Hospital, Ghent, Belgium**

^8^Department of Anesthesiology and Perioperative Medicine

**Città della Salute e dela Scienza, Turin, Italy**

^9^Department of Anesthesiology

**Uppsala University, Uppsala, Sweden**

^10^Department of Medical Sciences, Clinical Physiology

**Medical University Vienna, Vienna, Austria**

^12^Division of Cardiac, Thoracic, and Vascular Anesthesia and Intensive Care

^16^Department of Anaesthesia, Critical Care and Pain Medicine

**Saint Eloi University Hospital, Montpellier, France**

^13^Department of Critical Care Medicine and Anesthesiology (SAR B)

**St. Michael’s Hospital, University of Toronto, Toronto, Canada**

^14^Department of Anesthesiology

**University Hospitals of Geneva, Geneva, Switzerland**

^15^Division of Anesthesiology

**Tel-Aviv Medical Center, Sackler Medical School, Tel Aviv, Israel**

^17^Departmen of Anesthesia, Pain and Critical Care

**Sheffield Teaching Hospitals, University of Sheffield, Sheffield, United Kingdom**

^18^Operating Services, Critical Care and Anaesthesia (OSCCA)

**AZ Sint Jan Brugge-Oostende AV, Brugge, Belgium**

^19^Department of Anesthesiology

**University Hospital Bonn, Bonn, Germany**

^20^Department of Anesthesiology and Intensive Care Medicine

**Medical Faculty, RWTH Aachen University, Aachen, Germany**

^21^Department of Anesthesiology

**Istanbul University, Istanbul Faculty of Medicine, Istanbul, Turkey**

^23^Department of Anesthesiology and Intensive Care

**Universita’ dell'Insubria, Azienda Ospedaliera ASST Sette Laghi Ospedale di Circolo e Fondazione Macchi, Varese, Italy**

^24^Dipartimento di Anestesia

**Mayo Clinic, Rochester, Minnesota, USA**

^25^Department of Anesthesiology and Perioperative Medicine

**Massachusetts General Hospital, Boston, Massachusetts, USA**

^26^Department of Anesthesia, Critical Care and Pain Medicine

**University of Leipzig, Leipzig, Germany**

^27^Department of Anesthesiology and Intensive Care Medicine

**Bergmannstrost Hospital Halle, Halle, Germany**

^28^Department of Anaesthesiology, Intensive Care and Emergency Medicine, Pain Therapy

**Mahidol University, Bangkok, Thailand**

^29^Mahidol Oxford Tropical Medicine Research Unit (MORU), Faculty of Tropical Medicine

**^30^IRCCS San Martino Policlinico Hospital, Genoa, Italy**

**University of Genoa, Genoa, Italy**

^31^Department of Surgical Sciences and Integrated Diagnostics (DISC)

*Investigators*

| (**, indicates local principal investigators; names are listed in alphabetical order*) | |
| --- | --- |
| Abelha, Fernando* | Centro Hospitalar de São João, Department of Anaesthesiology, Porto, Portugal and Department of Surgery and Physiology, Cardiovascular Research Center, Faculty of Medicine of the University of Porto, Porto, Portugal |
| Abitağaoğlu, Sühayla | Fatih Sultan Mehmet Educational and Research Hospital, İstanbul, Turkey |
| Achilles, Marc* | Marienhospital Wesel, Wesel, Germany |
| Adebesin, Afeez | Imperial College Healthcare NHS Trust, UK |
| Adriaensens, Ine | University Hospital Antwerp, Belgium |
| Ahene, Charles* | Cleveland Clinic Abu Dhabi, Al Maryah Island, Abu Dhabi, UAE |
| Akbar, Fatima | Imperial College Healthcare NHS Trust, UK |
| Al Harbi, Mohammed | Ministry of National Guard Health Affairs, King Abdulaziz Medical City – Riyadh, Anesthesia Department and King Saud Bin Abdulaziz University for Health science, Saudi Arabia |
| Al Khoury al Kallab, Rita | Hôpitaux Universitaires de Genève, Département APSI, service d'anesthésiologie, Switzerland |
| Albanel, Xavier | Hôpitaux Universitaires de Genève, Département APSI, service d'anesthésiologie, Switzerland |
| Aldenkortt, Florence | Hôpitaux Universitaires de Genève, Département APSI, service d'anesthésiologie, Switzerland |
| Alfouzan, Rawan Abdullah Saleh | Ministry of National Guard Health Affairs, King Abdulaziz Medical City – Riyadh, Anesthesia Department, Saudi Arabia |
| Alruqaie, Reef | Ministry of National Guard Health Affairs, King Abdulaziz Medical City – Riyadh, Anesthesia Department, Saudi Arabia |
| Altermatt, Fernando* | Department of Anesthesiology. School of Medicine. Pontificia Universidad Catolica de Chile |
| Araujo, Bruno Luís de Castro* | Department of Anesthesiology, Hospital do Câncer II, National Cancer Institute of Brazil (INCA), Rio de Janeiro, Brazil |
| Arbesú, Genaro | University Hospital Germans Trias i Pujol, ICS, UAB, Spain |
| Artsi, Hanna | Division of Anesthesia, Pain and Critical Care, Tel-Aviv Medical Center affiliated with Sackler Medical School, Tel Aviv University, Tel Aviv, Israel |
| Aurilio, Caterina* | Department of Women, child and General and Specialized Surgery. University of Campania "L. Vanvitelli", Italy. |
| Ayanoglu, Omer Hilmi | Marmara University Pendik Training and Research Hospital, Turkey |
| Bacuzzi, Alessandro | Universita’ dell’Insubria – Dipartimento di Anestesia - Azienda Ospedaliera Asst Settlaghi Ospedale di Circolo e Fondazione Macchi – 21100 Varese - Italy |
| Baig, Harris* | University of Mississippi Medical Center, MS, USA |
| Baird, Yolanda | St Richard’s Hospital, Chichester, UK |
| Balonov, Konstantin* | Department of Anesthesiology and Perioperative Medicine, Tufts Medical Center, Boston, MA, USA |
| Balust, Jaume | Department of Anaesthesiology, Hospital Clínic, Barcelona, Spain. |
| Banks, Samantha | Royal Cornwall Hospital NHS Trust, UK |
| Bao, Xiaodong | Department of Anesthesia, Critical Care and Pain Medicine, Massachusetts General Hospital, Boston, Massachusetts, USA |
| Baumgartner, Mélanie | Hôpitaux Universitaires de Genève, Département APSI, service d'anesthésiologie, Switzerland |
| Belda Tortosa, Isabel* | Hospital Sagrat Cor- Barcelona, Spain |
| Bergamaschi, Alice | Department of Anesthesiology and Intensive Care, Pulmonary Engineering Group, University Hospital Carl Gustav Carus, Technische Universität Dresden, Germany |
| Bergmann, Lars* | Klinik für Anästhesiologie, Intensivmedizin und Schmerztherapie, Universitätsklinikum Knappschaftskrankenhaus Bochum, Bochum, Germany |
| Bigatello, Luca* | St. Elizabeth's Medical Center, MA, USA |
| Biosca Pérez, Elena | Consorcio Hospital General Universitario of Valencia, Spain |
| Birr, Katja | Department of Anesthesiology and Intensive Care, Pulmonary Engineering Group, University Hospital Carl Gustav Carus, Technische Universität Dresden, Germany |
| Bluth, Thomas | Department of Anesthesiology and Intensive Care, Pulmonary Engineering Group, University Hospital Carl Gustav Carus, Technische Universität Dresden, Germany |
| Bojaxhi, Elird | Department of Anesthesiology and Perioperative Medicine, Mayo Clinic Jacksonville, USA |
| Bonenti, Chiara | Universita’ dell’Insubria – Dipartimento di Anestesia - Azienda Ospedaliera Asst Settlaghi Ospedale di Circolo e Fondazione Macchi – 21100 Varese - Italy |
| Bonney, Iwona | Department of Anesthesiology and Perioperative Medicine, Tufts Medical Center, Boston, MA, USA |
| Bos, Elke M.E. | Amsterdam UMC, University of Amsterdam, Department of Anesthesiology, Meibergdreef 9, Amsterdam, The Netherlands |
| Bowman, Sara | Homerton University Hospitals NHS Foundation Trust, UK |
| Braz, Leandro Gobbo | Department of Anesthesiology, Universidade Estadual Paulista, Botucatu/SP, Brazil |
| Brugnoni, Elisa | Universita’ dell’Insubria – Dipartimento di Anestesia - Azienda Ospedaliera Asst Settlaghi Ospedale di Circolo e Fondazione Macchi – 21100 Varese - Italy |
| Brull, Sorin J | Department of Anesthesiology and Perioperative Medicine, College of Medicine, Mayo Clinic Jacksonville, FL, USA |
| Brunetti, Iole | Anestesia e Terapia Intensiva - IRCCS Policlinico San Martino – Genova – Italy |
| Bruni, Andrea | Anesthesia and Intensive Care, Department of Medical and Surgical Sciences, Magna Graeca University, Catanzaro, Italy |
| Buenvenida, Shonie L. | Department of Anesthesiology and Perioperative Medicine, Mayo Clinic, Rochester, Minnesota, USA |
| Busch, Cornelius Johannes* | Department of Anesthesiology, Heidelberg University Hospital, Im Neuenheimer Feld 110, 69120 Heidelberg, Germany |
| Camerini, Giovanni | Dipartimento di Scienze Chirurgiche e Diagnostiche Integrate – DISC – Università degli Studi di Genova – Genova – Italy and Clinica Chirurgica 1 - IRCCS Policlinico San Martino – Genova – Italy |
| Canet, Jaume* | University Hospital Germans Trias i Pujol, ICS, UAB, Spain |
| Capatti, Beatrice | Department Morphology, Surgery and Experimental Medicine. Anesthesia and intensive care universitary section. University of Ferrara. Italy |
| Carmona, Javiera | Department of Anesthesiology. School of Medicine. Pontificia Universidad Catolica de Chile |
| Carungcong, Jaime | Magill Department of Anaesthesia, Chelsea & Westminster NHS Foundation Trust, 369 Fulham Road, London, SW10 9NH, UK |
| Carvalho, Marta | Serviço de Anestesiologia, Centro Hospitalar do Porto, Porto, Portugal |
| Cattan, Anat | Division of Anesthesia, Pain and Critical Care, Tel-Aviv Medical Center affiliated with Sackler Medical School, Tel Aviv University, Tel Aviv, Israel |
| Cavaleiro, Carla | Serviço de Anestesiologia, Centro Hospitalar do Porto, Porto, Portugal and Centro de Investigação Clínica em Anestesiologia, Serviço de Anestesiologia, Centro Hospitalar do Porto, Porto, Portugal |
| Chiumello, Davide* | Dipartimento di Scienze della Salute, Università degli Studi di Milano, Milan, Italy and SC Anestesia e Rianimazione, ASST Santi Paolo e Carlo, Milan, Italy, Centro ricerca cordinata insufficienza respiratoria |
| Ciardo, Stefano | Department Morphology, Surgery and Experimental Medicine. Anesthesia and intensive care universitary section. University of Ferrara. Italy |
| Coburn, Mark | Department of Anesthesiology, Medical Faculty, RWTH Aachen University, Aachen, Germany |
| Colella, Umberto | Department of Women, child and General and Specialized Surgery. University of Campania "L. Vanvitelli", Italy. |
| Contreras, Victor | Department of Anesthesiology. School of Medicine. Pontificia Universidad Catolica de Chile |
| Corman Dincer, Pelin* | Marmara University Pendik Training and Research Hospital, Turkey |
| Cotter, Elizabeth | Section of Critical Care Medicine, Department of Anesthesia and Critical Care, University of Chicago, Chicago, IL, USA |
| Crovetto, Marcia | Department of Anesthesiology. School of Medicine. Pontificia Universidad Catolica de Chile |
| Darrah, William | Department of Anesthesiology, St. Michael’s Hospital, University of Toronto, Toronto, ON, Canada |
| Davies, Simon* | York Teaching Hospitals NHS Foundation Trust, UK |
| de Baerdemaeker, Luc* | Ghent University Hospital, Ghent, Belgium. Corneel Heymanslaan 10, 9000 Gent, Belgium |
| De Hert, Stefan | Ghent University Hospital, Ghent, Belgium. Corneel Heymanslaan 10, 9000 Gent, Belgium |
| Del Cojo Peces, Enrique | Área de Salud Don Benito-Villanueva/Don Benito-Villanueva Health Area, Spain. |
| Delphin, Ellise | Montefiore Medical Center, Bronx, NY 10467, USA |
| Diaper, John | Hôpitaux Universitaires de Genève, Département APSI, service d'anesthésiologie, Switzerland |
| do Nascimento Junior, Paulo* | Department of Anesthesiology, Universidade Estadual Paulista, Botucatu/SP, Brazil |
| Donatiello, Valerio | Department of Women, child and General and Specialized Surgery. University of Campania "L. Vanvitelli", Italy. |
| Dong, Jing | Department of Anesthesiology, Fudan University Shanghai Cancer Center, Shanghai, China. Department of Oncology, Shanghai Medical College, Fudan University, Shanghai, China |
| Dourado, Maria do Socorro | Associação Hospitalar Beneficente São Vicente de Paulo, Brazil |
| Dullenkopf, Alexander* | Frauenfeld Cantonal Hospital, Switzerland |
| Ebner, Felix | Klinik für Anästhesiologie, Intensivmedizin und Schmerztherapie,Universitätsklinikum Knappschaftskrankenhaus Bochum, Bochum, Germany |
| Elgendy, Hamed* | Departments of Anesthesiology, King Abdullah Medical City, Makkah, Saudi Arabia and Assiut University, Egypt and Hamad Medical Corporation, Qatar. |
| Ellenberger, Christoph | Hôpitaux Universitaires de Genève, Département APSI, service d'anesthésiologie, Switzerland |
| Erdoğan Arı, Dilek* | Fatih Sultan Mehmet Educational and Research Hospital, İstanbul, Turkey |
| Ermert, Thomas | Department of Anesthesiology, Intensive Care and Pain Medicine, University Hospital Münster, Münster, Germany |
| Farah, Fadi | St. Elizabeth's Medical Center, MA, USA |
| Fernandez-Bustamante, Ana* | University of Colorado School of Medicine, Department of Anesthesiology, CO, USA |
| Ferreira, Cristina | Serviço de Anestesiologia, Centro Hospitalar do Porto, Porto, Portugal |
| Fiore, Marco | Department of Women, child and General and Specialized Surgery. University of Campania "L. Vanvitelli", Italy. |
| Fonte, Ana | Department of Anesthesiology from Centro Hospitalar de Entre o Douro e Vouga, EPE – São Sebastião Hospital, Santa Maria da Feira, Portugal |
| Fortià Palahí, Christina | Hospital Sagrat Cor- Barcelona, Spain |
| Galimberti, Andrea | Dipartimento di Scienze della Salute, Università degli Studi di Milano, Milan, Italy and SC Anestesia e Rianimazione, Asst Santi Paolo e Carlo, Milan, Italy, Centro ricerca cordinata insufficienza respiratoria |
| Gama de Abreu, Marcelo* | Department of Anesthesiology and Intensive Care, Pulmonary Engineering Group, University Hospital Carl Gustav Carus, Technische Universität Dresden, Germany |
| Garofano, Najia | Hôpitaux Universitaires de Genève, Département APSI, service d'anesthésiologie, Switzerland |
| Giaccari, Luca Gregorio | Department of Women, child and General and Specialized Surgery. University of Campania "L. Vanvitelli", Italy. |
| Gilsanz, Fernando | Department of Anesthesiology and Surgical Critical Care, Hospital Universitario La Paz, Madrid, Spain |
| Girrbach, Felix | Department of Anaesthesiology and Intensive Care Medicine, University of Leipzig, Leipzig, Germany; |
| Gobbi, Luca | Department Morphology, Surgery and Experimental Medicine. Anesthesia and intensive care universitary section. University of Ferrara. Italy |
| Godfried,Marc Bernard* | Department of Anesthesiology, Onze Lieve Vrouwe Gasthuis, Amsterdam, The Netherlands |
| Goettel, Nicolai* | Department of Anesthesia, Surgical Intensive Care, Prehospital Emergency Medicine and Pain Therapy, University Hospital Basel, University of Basel, Basel, Switzerland |
| Goldstein, Peter A.* | Weill Cornell Medicine, New York, NY, USA |
| Goren, Or | Division of Anesthesia, Pain and Critical Care, Tel-Aviv Medical Center affiliated with Sackler Medical School, Tel Aviv University, Tel Aviv, Israel |
| Gorlin, Andrew | Mayo Clinic Arizona, 5777 East Mayo Blvd, Phoenix, AZ, 85054, USA |
| Granell Gil, Manuel* | Consorcio Hospital General Universitario of Valencia, Spain |
| Gratarola, Angelo | Anestesia e Rianimazione - IRCCS Policlinico San Martino – Genova – Italy |
| Graterol, Juan* | Royal Cornwall Hospital NHS Trust, UK |
| Guyon, Pierre | Hôpitaux Universitaires de Genève, Département APSI, service d'anesthésiologie, Switzerland |
| Haire, Kevin | Magill Department of Anaesthesia, Chelsea & Westminster NHS Foundation Trust, 369 Fulham Road, London, SW10 9NH, UK |
| Harou, Philippe | Polyclinique Montier la Celle, France |
| Helf, Antonia | Department of Anaesthesiology, University Hospital Würzburg, Würzburg, Germany |
| Hemmes, Sabrine N. T.* | Amsterdam UMC, University of Amsterdam, Department of Anesthesiology, Meibergdreef 9, Amsterdam, The Netherlands |
| Hempel, Gunther | Department of Anaesthesiology and Intensive Care Medicine, University of Leipzig, Leipzig, Germany; |
| Hernández Cádiz, María José | Consorcio Hospital General Universitario of Valencia, Spain |
| Heyse, Björn | Ghent University Hospital, Ghent, Belgium. Corneel Heymanslaan 10, 9000 Gent, Belgium |
| Hollmann, Markus W.* | Amsterdam UMC, University of Amsterdam, Department of Anesthesiology, Meibergdreef 9, Amsterdam, The Netherlands |
| Huercio, Ivan | Department of Anesthesiology and Surgical Critical Care, Hospital Universitario La Paz, Madrid, Spain |
| Ilievska, Jasmina | University Clinic of Surgery “Ss. Naum Ohridski”, Faculty of Medicine, University “Ss. Cyril and Methodius”, Skopje, Macedonia |
| Jakus, Lien | Hôpitaux Universitaires de Genève, Département APSI, service d'anesthésiologie, Switzerland |
| Jeganath, Vijay | Royal Stoke University Hospital, UHNM NHS Trust, Stoke-on-Trent, Staffordshire, ST4 6QG, UK |
| Jelting, Yvonne | Department of Anaesthesiology, University Hospital Würzburg, Würzburg, Germany |
| Jung, Minoa | Hôpitaux Universitaires de Genève, Département APSI, service d'anesthésiologie, Switzerland |
| Kabon, Barbara* | Department of Anaesthesia,Critical Care and Pain Medicine, Medical University Vienna, Austria |
| Kacha, Aalok* | Section of Critical Care Medicine, Department of Anesthesia and Critical Care, University of Chicago, Chicago, IL, USA |
| Karaman Ilić, Maja | Clinical hospital Sveti Duh, JJ Strossmayer, Faculty of Medicine, Zagreb, Croatia |
| Karuppiah, Arunthevaraja | St. Elizabeth's Medical Center, MA, USA |
| Kavas, Ayse Duygu | Marmara University Pendik Training and Research Hospital, Turkey |
| Keli Barcelos, Gleicy | Hôpitaux Universitaires de Genève, Département APSI, service d'anesthésiologie, Switzerland |
| Kellogg, Todd A. | Department of Surgery, Mayo Clinic, Rochester, Minnesota, USA |
| Kemper, Johann | Department of Anesthesiology, University Hospital Duesseldorf, Heinrich-Heine University Duesseldorf, Duesseldorf, Germany |
| Kerbrat, Romain | Hôpitaux Universitaires de Genève, Département APSI, service d'anesthésiologie, Switzerland |
| Khodr, Suraya | Cleveland Clinic Abu Dhabi, Al Maryah Island, Abu Dhabi, UAE |
| Kienbaum, Peter | Department of Anesthesiology, University Hospital Duesseldorf, Heinrich-Heine University Duesseldorf, Duesseldorf, Germany |
| Kir, Bunyamin | Marmara University Pendik Training and Research Hospital, Turkey |
| Kiss, Thomas | Department of Anesthesiology and Intensive Care, Pulmonary Engineering Group, University Hospital Carl Gustav Carus, Technische Universität Dresden, Germany |
| Kivrak, Selin | Hôpitaux Universitaires de Genève, Département APSI, service d'anesthésiologie, Switzerland |
| Klarić, Vlasta* | Clinic of anesthesiology, reanimatology and intensive care medicine, University Hospital Dubrava, Zagreb, Croatia |
| Koch, Thea | Department of Anesthesiology and Intensive Care, Pulmonary Engineering Group, University Hospital Carl Gustav Carus, Technische Universität Dresden, Germany |
| Köksal, Ceren | Fatih Sultan Mehmet Educational and Research Hospital, İstanbul, Turkey |
| Kowark, Ana | Department of Anesthesiology, Medical Faculty, RWTH Aachen University, Aachen, Germany |
| Kranke, Peter* | Department of Anaesthesiology, University Hospital Würzburg, Würzburg, Germany |
| Kuvaki, Bahar* | Dokuz Eylül University Faculty of Medicine, Department of Anaesthesiology and Intensive Care, Turkey |
| Kuzmanovska, Biljana | University clinic for Traumatology, Orthopedics, Anesthesia, Reanimation, Intensive care and Emmergency center - Skopje, Department of Anesthesiology, Reanimation and Intensive care Medicine - Skopje, Macedonia |
| Laffey, John* | Department of Anesthesiology, St. Michael’s Hospital, University of Toronto, Toronto, ON, Canada |
| Lange, Mirko | Department of Anaesthesiology and Intensive Care Medicine, University of Leipzig, Leipzig, Germany; |
| Lemos, Marília Freitas de | Department of Anesthesiology, Hospital do Câncer II, National Cancer Institute of Brazil (INCA), Rio de Janeiro, Brazil |
| Licker, Marc-Joseph* | Hôpitaux Universitaires de Genève, Département APSI, service d'anesthésiologie; Faculty of Medicine, University of Geneva, Switzerland |
| López-Baamonde, Manuel | Department of Anaesthesiology, Hospital Clínic, Barcelona, Spain. |
| López-Hernández, Antonio | Department of Anaesthesiology, Hospital Clínic, Barcelona, Spain. |
| Lopez-Martinez, Mercedes | Department of Anesthesiology and Surgical Critical Care, Hospital Universitario La Paz, Madrid, Spain |
| Luise, Stéphane | Hôpitaux Universitaires de Genève, Département APSI, service d'anesthésiologie, Switzerland |
| MacGregor, Mark* | Ashford and St Peters NHS Foundation Trust, UK |
| Magalhães, Danielle* | Associação Hospitalar Beneficente São Vicente de Paulo, Brazil |
| Maillard, Julien | Hôpitaux Universitaires de Genève, Département APSI, service d'anesthésiologie, Switzerland |
| Malerbi, Patrizia | Anestesia e Rianimazione - IRCCS Policlinico San Martino – Genova – Italy |
| Manimekalai, Natesan | University of Mississippi Medical Center, MS, USA |
| Margarson, Michael* | St Richard’s Hospital, Chichester, UK |
| Markstaller, Klaus* | Department of Anaesthesia,Critical Care and Pain Medicine, Medical University Vienna, Austria |
| Martin, Archer K | Department of Anesthesiology and Perioperative Medicine, College of Medicine, Mayo Clinic Jacksonville, FL, USA |
| Martin, David P. | Department of Anesthesiology and Perioperative Medicine, Mayo Clinic, Rochester, Minnesota, USA |
| Martin, Yvette N. | Department of Anesthesiology and Perioperative Medicine, Mayo Clinic, Rochester, Minnesota, USA |
| Martínez-Ocon, Julia | Department of Anaesthesiology, Hospital Clínic, Barcelona, Spain. |
| Martin-Loeches, Ignacio* | Department of Intensive Care Medicine, Multidisciplinary Intensive Care Research Organization (MICRO), St. James's Hospital, Dublin, Ireland |
| Maseda, Emilio | Department of Anesthesiology and Surgical Critical Care, Hospital Universitario La Paz, Madrid, Spain |
| Matot, Idit* | Division of Anesthesia, Pain and Critical Care, Tel-Aviv Medical Center affiliated with Sackler Medical School, Tel Aviv University, Tel Aviv, Israel |
| McAuliffe, Niamh | Department of Anesthesiology, St. Michael’s Hospital, University of Toronto, Toronto, ON, Canada |
| McKenzie, Travis J. | Department of Surgery, Mayo Clinic, Rochester, Minnesota, USA |
| Medina, Paulina | Department of Anesthesiology. School of Medicine. Pontificia Universidad Catolica de Chile |
| Meersch, Melanie | Department of Anesthesiology, Intensive Care and Pain Medicine, University Hospital Münster, Münster, Germany |
| Menzen, Angelika* | St. Marienhospital gGmbh Friesoythe, Friesoythe, Germany |
| Mertens, Els* | University Hospital Antwerp, Belgium |
| Meurer, Bernd | Marienhospital Wesel, Wesel, Germany |
| Meyer-Treschan, Tanja* | Department of Anesthesiology, University Hospital Duesseldorf, Heinrich-Heine University Duesseldorf, Duesseldorf, Germany |
| Miao, Changhong* | Department of Anesthesiology, Fudan University Shanghai Cancer Center, Shanghai, China. Department of Oncology, Shanghai Medical College, Fudan University, Shanghai, China |
| Micalizzi, Camilla | Dipartimento di Scienze Chirurgiche e Diagnostiche Integrate – DISC – Università degli Studi di Genova – Genova – Italy and Anestesia e Terapia Intensiva - IRCCS Policlinico San Martino – Genova – Italy |
| Milić, Morena | Clinic of anesthesiology, reanimatology and intensive care medicine, University Hospital Dubrava, Zagreb, Croatia |
| Módolo, Norma Sueli Pinheiro | Department of Anesthesiology, Universidade Estadual Paulista, Botucatu/SP, Brazil |
| Moine, Pierre | University of Colorado School of Medicine, Department of Anesthesiology, CO, USA |
| Mölders, Patrick | Klinik für Anästhesiologie, Intensivmedizin und Schmerztherapie,Universitätsklinikum Knappschaftskrankenhaus Bochum, Bochum, Germany |
| Montero-Feijoo, Ana | Department of Anesthesiology and Surgical Critical Care, Hospital Universitario La Paz, Madrid, Spain |
| Moret, Enrique | University Hospital Germans Trias i Pujol, ICS, UAB, Spain |
| Muller, Markus K. | Frauenfeld Cantonal Hospital, Switzerland |
| Murphy, Zoe | York Teaching Hospitals NHS Foundation Trust, UK |
| Nalwaya, Pramod* | Royal Stoke University Hospital, UHNM NHS Trust, Stoke-on-Trent, Staffordshire, ST4 6QG, UK |
| Naumovski, Filip* | University clinic for Traumatology, Orthopedics, Anesthesia, Reanimation, Intensive care and Emmergency center - Skopje, Department of Anesthesiology, Reanimation and Intensive care Medicine - Skopje, Macedonia |
| Navalesi, Paolo* | Anesthesia and Intensive Care, Department of Medical and Surgical Sciences, Magna Graeca University, Catanzaro, Italy |
| Navarro e Lima, Lais Helena | Department of Anesthesiology, Universidade Estadual Paulista, Botucatu/SP, Brazil |
| Nesek Adam, Višnja* | Clinical hospital Sveti Duh, JJ Strossmayer, Faculty of Medicine, Zagreb, Croatia |
| Neumann, Claudia | Department of Anaesthesiology and Intensive Care Medicine, University Hospital of Bonn, Bonn, Germany |
| Newell, Christopher* | Southmead Hospital, North Bristol NHS Trust, UK |
| Nisnevitch, Zoulfira* | Montefiore Medical Center, Bronx, NY 10467, USA |
| Nizamuddin, Junaid | Section of Critical Care Medicine, Department of Anesthesia and Critical Care, University of Chicago, Chicago, IL, USA |
| Novazzi, Cecilia | Universita’ dell’Insubria – Dipartimento di Anestesia - Azienda Ospedaliera Asst Settlaghi Ospedale di Circolo e Fondazione Macchi – 21100 Varese - Italy |
| O'Connor, Michael | Section of Critical Care Medicine, Department of Anesthesia and Critical Care, University of Chicago, Chicago, IL, USA |
| Oprea, Günther | Klinik für Anästhesiologie, Intensivmedizin und Schmerztherapie, Universitätsklinikum Knappschaftskrankenhaus Bochum, Bochum, Germany |
| Orhan Sungur, Mukadder | Istanbul University, Istanbul Faculty of Medicine, Turkey |
| Özbilgin, Şule | Dokuz Eylül University Faculty of Medicine, Department of Anaesthesiology and Intensive Care, Turkey |
| Pace, Maria Caterina | Department of Women, child and General and Specialized Surgery. University of Campania "L. Vanvitelli", Italy. |
| Pacheco, Marcos | Department of Anesthesiology from Centro Hospitalar de Entre o Douro e Vouga, EPE – São Sebastião Hospital, Santa Maria da Feira, Portugal |
| Packianathaswamy, Balaji* | Hull and East Yorkshire Hospitals NHS Trust, UK |
| Palma Gonzalez, Estefania | Área de Salud Don Benito-Villanueva/Don Benito-Villanueva Health Area, Spain. |
| Papaspyros, Fotios* | POLYCLINIQUE MONTIER LA CELLE, France |
| Paredes, Sebastián | Department of Anesthesiology. School of Medicine. Pontificia Universidad Catolica de Chile |
| Passavanti, Maria Beatrice | Department of Women, child and General and Specialized Surgery. University of Campania "L. Vanvitelli", Italy. |
| Pedemonte, Juan Cristobal | Department of Anesthesiology. School of Medicine. Pontificia Universidad Catolica de Chile |
| Pelosi, Paolo* | Dipartimento di Scienze Chirurgiche e Diagnostiche Integrate – DISC – Università degli Studi di Genova – Genova – Italy and Anestesia e Terapia Intensiva - IRCCS Policlinico San Martino – Genova – Italy |
| Peremin, Sanja | Clinic of anesthesiology, reanimatology and intensive care medicine, University Hospital Dubrava, Zagreb, Croatia |
| Philipsenburg, Christoph | Department of Anesthesiology, Heidelberg University Hospital, Im Neuenheimer Feld 110, 69120 Heidelberg, Germany |
| Pinho, Daniela | Serviço de Anestesiologia, Centro Hospitalar do Porto, Porto, Portugal |
| Pinho, Silvia | Serviço de Anestesiologia, Centro Hospitalar do Porto, Porto, Portugal |
| Posthuma, Linda M. | Amsterdam UMC, University of Amsterdam, Department of Anesthesiology, Meibergdreef 9, Amsterdam, The Netherlands |
| Pota, Vincenzo | Department of Women, child and General and Specialized Surgery. University of Campania "L. Vanvitelli", Italy. |
| Preckel, Benedikt | Amsterdam UMC, University of Amsterdam, Department of Anesthesiology, Meibergdreef 9, Amsterdam, The Netherlands |
| Priani, Paolo | Department Morphology, Surgery and Experimental Medicine. Anesthesia and intensive care universitary section. University of Ferrara. Italy |
| Putensen, Christian* | Department of Anaesthesiology and Intensive Care Medicine, University Hospital of Bonn, Bonn, Germany |
| Rached, Mohamed Aymen | Hôpitaux Universitaires de Genève, Département APSI, service d'anesthésiologie, Switzerland |
| Radoeshki, Aleksandar | University Clinic of Surgery “Ss. Naum Ohridski”, Faculty of Medicine, University “Ss. Cyril and Methodius”, Skopje, Macedonia |
| Ragazzi, Riccardo | Department Morphology, Surgery and Experimental Medicine. Anesthesia and intensive care universitary section. University of Ferrara. Italy |
| Rajamanickam, Tamilselvan | Royal Stoke University Hospital, UHNM NHS Trust, Stoke-on-Trent, Staffordshire, ST4 6QG, UK |
| Rajamohan, Arthi | Department of Anesthesiology, St. Michael’s Hospital, University of Toronto, Toronto, ON, Canada |
| Ramakrishna, Harish* | Mayo Clinic Arizona, 5777 East Mayo Blvd, Phoenix, AZ, 85054, USA |
| Rangarajan, Desikan* | Homerton University Hospitals NHS Foundation Trust, UK |
| Reiterer, Christian | Department of Anaesthesia,Critical Care and Pain Medicine, Medical University Vienna, Austria |
| Renew, J. Ross | Department of Anesthesiology and Perioperative Medicine, Mayo Clinic Jacksonville, USA |
| Reynaud, Thomas | Hôpitaux Universitaires de Genève, Département APSI, service d'anesthésiologie, Switzerland |
| Rhys, Rhidian | Southmead Hospital, North Bristol NHS Trust, UK |
| Rivas, Eva* | Department of Anaesthesiology, Hospital Clínic, IDIBAPS, Universitat de Barcelona, Spain |
| Robitzky, Luisa | Klinik für Anästhesiologie, Intensivmedizin und Schmerztherapie, Universitätsklinikum Knappschaftskrankenhaus Bochum, Bochum, Germany |
| Rossaint, Rolf* | Department of Anesthesiology, Medical Faculty, RWTH Aachen University, Aachen, Germany |
| Rubulotta, Francesca* | Imperial College Healthcare NHS Trust, UK |
| S. Machado, Humberto* | Serviço de Anestesiologia, Centro Hospitalar do Porto, Porto, Portugal and Instituto Ciências Biomédicas Abel Salazar, Universidade do Porto, Porto, Portugal and Centro de Investigação Clínica em Anestesiologia, Serviço de Anestesiologia, Centro Hospitalar do Porto, Porto, Portugal |
| S. Nunes, Catarina | Universidade Aberta, Departamento de Ciências e Tecnologia, Porto, Portugal and Centro de Investigação Clínica em Anestesiologia, Serviço de Anestesiologia, Centro Hospitalar do Porto, Porto, Portugal |
| Sabbatini, Giovanni | Dipartimento di Scienze della Salute, Università degli Studi di Milano, Milan, Italy and SC Anestesia e Rianimazione, ASST Santi Paolo e Carlo, Milan, Italy, Centro ricerca cordinata insufficienza respiratoria |
| Samuels, Jon D | Weill Cornell Medicine, Department of Anesthesiology, New York-Presbyterian Hospital, New York, NY, USA |
| Sanahuja, Josep Martí | Department of Anaesthesiology, Hospital Clínic, Barcelona, Spain. |
| Sansone, Pasquale | Department of Women, child and General and Specialized Surgery. University of Campania "L. Vanvitelli", Italy. |
| Santos, Alice | Department of Anaesthesiology, Centro Hospitalar São João, Porto, Portugal |
| Sayedalahl, Mohamed | Departments of Anesthesiology, King Abdullah Medical City, Makkah, Saudi Arabia and Mansoura University, Egypt |
| Schaefer, Maximilian S. | Department of Anesthesiology, University Hospital Duesseldorf, Heinrich-Heine University Duesseldorf, Duesseldorf, Germany |
| Scharffenberg, Martin | Department of Anesthesiology and Intensive Care, Pulmonary Engineering Group, University Hospital Carl Gustav Carus, Technische Universität Dresden, Germany |
| Schiffer, Eduardo | Hôpitaux Universitaires de Genève, Département APSI, service d'anesthésiologie; Faculty of Medicine, University of Geneva, Switzerland |
| Schliewe, Nadja | Department of Anaesthesiology and Intensive Care Medicine, University of Leipzig, Leipzig, Germany; |
| Schorer, Raoul | Hôpitaux Universitaires de Genève, Département APSI, service d'anesthésiologie, Switzerland |
| Schultz, Marcus J. | Amsterdam UMC, University of Amsterdam, Department of Anesthesiology, Meibergdreef 9, Amsterdam, The Netherlands |
| Schumann, Roman | Department of Anesthesiology and Perioperative Medicine, Tufts Medical Center, Boston, MA, USA |
| Selmo, Gabriele | Universita’ dell’Insubria – Dipartimento di Anestesia - Azienda Ospedaliera Asst Settlaghi Ospedale di Circolo e Fondazione Macchi – 21100 Varese - Italy |
| Sendra, Mar | University Hospital Germans Trias i Pujol, ICS, UAB, Spain |
| Senturk, Mert* | Istanbul University, Istanbul Faculty of Medicine, Turkey |
| Severgnini, Paolo* | Universita’ dell’Insubria – Dipartimento di Anestesia - Azienda Ospedaliera Asst Settlaghi Ospedale di Circolo e Fondazione Macchi – 21100 Varese - Italy |
| Shaw, Kate | York Teaching Hospitals NHS Foundation Trust, UK |
| Shosholcheva, Mirjana* | University Clinic of Surgery “Ss. Naum Ohridski”, Faculty of Medicine, University “Ss. Cyril and Methodius”, Skopje, Macedonia |
| Sibai, Abdulrazak* | MINISTRY OF NATIONAL GUARD HEALTH AFFAIRS, KING ABDULAZIZ MEDICAL CITY – RIYADH, Anesthesia Department, Saudi Arabia |
| Simon, Philipp | Department of Anaesthesiology and Intensive Care Medicine, University of Leipzig, Leipzig, Germany; |
| Simonassi, Francesca | Dipartimento di Scienze Chirurgiche e Diagnostiche Integrate – DISC – Università degli Studi di Genova – Genova – Italy and Anestesia e Terapia Intensiva - IRCCS Policlinico San Martino – Genova – Italy |
| Sinno, Claudia | Universita’ dell’Insubria – Dipartimento di Anestesia - Azienda Ospedaliera Asst Settlaghi Ospedale di Circolo e Fondazione Macchi – 21100 Varese - Italy |
| Sivrikoz, Nukhet | Istanbul University, Istanbul Faculty of Medicine, Turkey |
| Skandalou, Vasiliki* | Alexandra General Hospital, Athens, Greece |
| Smith, Neil | Hull and East Yorkshire Hospitals NHS Trust, UK |
| Soares, Maria | Serviço de Anestesiologia, Centro Hospitalar do Porto, Porto, Portugal |
| Socorro Artiles, Tania | University Clinic Hospital Valencia, Spain |
| Sousa Castro, Diogo* | Department of Anesthesiology from Centro Hospitalar de Entre o Douro e Vouga, EPE – São Sebastião Hospital, Santa Maria da Feira, Portugal |
| Sousa, Miguel | Department of Anesthesiology from Centro Hospitalar de Entre o Douro e Vouga, EPE – São Sebastião Hospital, Santa Maria da Feira, Portugal |
| Spadaro, Savino* | Department Morphology, Surgery and Experimental Medicine. Anesthesia and intensive care universitary section. University of Ferrara. Italy |
| Sprung, Juraj* | Department of Anesthesiology and Perioperative Medicine, Mayo Clinic, Rochester, Minnesota, USA |
| Stamatakis, Emmanouil | Alexandra General Hospital, Athens, Greece |
| Steiner, Luzius A. | Department of Anesthesia, Surgical Intensive Care, Prehospital Emergency Medicine and Pain Therapy, University Hospital Basel, University of Basel, Basel, Switzerland |
| Stevenazzi, Andrea | University Hospital Germans Trias i Pujol, ICS, UAB, Spain |
| Suarez-de-la-Rica, Alejandro* | Department of Anesthesiology and Surgical Critical Care, Hospital Universitario La Paz, Madrid, Spain |
| Suppan, Mélanie | Hôpitaux Universitaires de Genève, Département APSI, service d'anesthésiologie, Switzerland |
| Teichmann, Robert | Department of Anesthesiology and Intensive Care, Pulmonary Engineering Group, University Hospital Carl Gustav Carus, Technische Universität Dresden, Germany |
| Tena Guerrero, José Maria* | Área de Salud Don Benito-Villanueva/Don Benito-Villanueva Health Area, Spain. |
| Thiel, Bram | Department of Anesthesiology, Onze Lieve Vrouwe Gasthuis, Amsterdam, The Netherlands |
| Tolós, Raquel | University Hospital Germans Trias i Pujol, ICS, UAB, Spain |
| Tore Altun, Gulbin | Marmara University Pendik Training and Research Hospital, Turkey |
| Tucci, Michelle | University of Mississippi Medical Center, MS, USA |
| Turnbull, Zachary A. | Weill Cornell Medicine, New York, NY, USA |
| Turudić, Žana | Clinic of anesthesiology, reanimatology and intensive care medicine, University Hospital Dubrava, Zagreb, Croatia |
| Unterberg, Matthias | Klinik für Anästhesiologie, Intensivmedizin und Schmerztherapie, Universitätsklinikum Knappschaftskrankenhaus Bochum, Bochum, Germany |
| Van Limmen, Jurgen | Ghent University Hospital, Ghent, Belgium. Corneel Heymanslaan 10, 9000 Gent, Belgium |
| Van Nieuwenhove, Yves | Ghent University Hospital, Ghent, Belgium. Corneel Heymanslaan 10, 9000 Gent, Belgium |
| Van Waesberghe, Julia | Department of Anesthesiology, Medical Faculty, RWTH Aachen University, Aachen, Germany |
| Vidal Melo, Marcos Francisco* | Department of Anesthesia, Critical Care and Pain Medicine, Massachusetts General Hospital, Boston, Massachusetts, USA |
| Vitković, Bibiana | Clinic of anesthesiology, reanimatology and intensive care medicine, University Hospital Dubrava, Zagreb, Croatia |
| Vivona, Luigi | Department of Anesthesiology and Intensive Care, Pulmonary Engineering Group, University Hospital Carl Gustav Carus, Technische Universität Dresden, Germany |
| Vizcaychipi, Marcela* | Magill Department of Anaesthesia, Chelsea & Westminster NHS Foundation Trust, 369 Fulham Road, London, SW10 9NH, UK |
| Volta, Carlo Alberto | Department Morphology, Surgery and Experimental Medicine. Anesthesia and intensive care universitary section. University of Ferrara. Italy |
| Weber, Anne | Hôpitaux Universitaires de Genève, Département APSI, service d'anesthésiologie, Switzerland |
| Weingarten, Toby N. | Department of Anesthesiology and Perioperative Medicine, Mayo Clinic, Rochester, Minnesota, USA |
| Wittenstein, Jakob | Department of Anesthesiology and Intensive Care, Pulmonary Engineering Group, University Hospital Carl Gustav Carus, Technische Universität Dresden, Germany |
| Wrigge, Hermann* | Department of Anaesthesiology and Intensive Care Medicine, University of Leipzig, Leipzig, Germany and Department of Anaesthesiology, Intensive Care and Emergency Medicine, Pain Therapy; Bergmannstrost Hospital Halle, Halle, Germany |
| Wyffels, Piet | Ghent University Hospital, Ghent, Belgium. Corneel Heymanslaan 10, 9000 Gent, Belgium |
| Yagüe, Julio | Department of Anesthesiology and Surgical Critical Care, Hospital Universitario La Paz, Madrid, Spain |
| Yates, David | York Teaching Hospitals NHS Foundation Trust, UK |
| Yavru, Ayşen | Istanbul University, Istanbul Faculty of Medicine, Turkey |
| Zac, Lilach | Division of Anesthesia, Pain and Critical Care, Tel-Aviv Medical Center affiliated with Sackler Medical School, Tel Aviv University, Tel Aviv, Israel |
| Zhong, Jing | Department of Anesthesiology, Fudan University Shanghai Cancer Center, Shanghai, China. Department of Oncology, Shanghai Medical College, Fudan University, Shanghai, China |

*Information and funding*

The Clinical Trials Network of the European Society of Anaesthesiology provided financial support for the steering committee meetings, onsite visits to participating sites, for the building of the electronic data capture system, and for the advertising of the study. The Technische Universität Dresden provided logistical support for the coordinating site. The Conselho Nacional de Desenvolvimento Científico e Tecnológico provided financial support for insurance in Brazil. The Association of Anaesthetists of Great Britain and Ireland and the Northern Ireland Society of Anaesthetists provided financial support for the participating sites in the United Kingdom.
