# Supplementary material for: Individual and combined effects of chemical and mechanical power on postoperative pulmonary complications: a secondary analysis of the REPEAT study
Source: Anaesthesia. 2025 Aug 19;80(12):1510–8. doi: 10.1111/anae.16725 (PMC12614417; doi:10.1111/anae.16725)
Supplement: Supplementary file 2 — Table S1. Definitions of postoperative pulmonary complications. Table S2. Patient characteristics (including only patients with available plateau pressures). Table S3. Intra‐operative ventilation parameters (including only patients with available plateau pressures). Table S4. Multivariable regression model to assess the associations of chemical and mechanical power with postoperative pulmonary complications (including only patients with available plateau pressures). Table S5. Patient characteristics (including only patients with FIO2 of 0.4, 0.5 or 0.8). Table S6. Intra‐operative ventilation parameters (including only patients with FIO2 of 0.4, 0.5 or 0.8). Table S7. Multivariable regression model to assess the associations of chemical and mechanical power with postoperative pulmonary complications (including only patients with FIO2 of 0.4, 0.5 or 0.8). Table S8. Patient characteristics (including all patients). Table S9. Intra‐operative ventilation parameters (including all patients). Table S10. Multivariable regression model to assess the associations of chemical and mechanical power with postoperative pulmonary complications (including all patients). Table S11. Multivariable regression model to assess the associations of chemical and mechanical power with postoperative pulmonary complications (adjustment of the primary analysis for trial effects). [file ANAE-80-1510-s004.docx]

***Appendix S2: Supplemental Tables***

**Table S1.** Definitions of postoperative pulmonary complications.

|  | **PROVHILO** | **iPROVE** | **PROBESE** |
| --- | --- | --- | --- |
| Mild respiratory failure | PaO2 < 60 mmHg or SpO2 < 90% breathing at least 10 minutes of room air but responding to supplemental oxygen of 2 l.min^-1^ | SpO2 < 92% with FiO2 of 0.21 or SpO2 < 95% with FiO2 of 0.50 | PaO2 < 60 mmHg or SpO2 < 90% breathing at least 10 minutes of room air but responding to supplemental oxygen of 2 l.min^-1^ |
| Severe respiratory failure | PaO2 < 60 mmHg or SpO2 < 90% breathing ≥ 10 minutes of room air but responding only to supplemental oxygen > 2 l.min^-1^ or need for noninvasive or invasive mechanical ventilation | Increased FiO2, increased requirement for CPAP, or the need for noninvasive or invasive ventilation | PaO2< 60 mmHg or SpO2 < 90% breathing ≥ 10 minutes of room air but responding only to supplemental oxygen > 2 l.min^-1^ or need for noninvasive or invasive mechanical ventilation |
| ARDS | AECC criteria* | Berlin criteria** | Berlin criteria** |
| Pulmonary infection | Need of antibiotics and at least one of the following criteria: new or changed sputum, new or changed lung opacities on chest X-ray when clinically indicated, tympanic temperature >38.3°C, WBC count >12,000.μl^-1^ in the absence of other infectious focus | Presence of a new pulmonary infiltrate and/or progression of previous pulmonary infiltrates on a chest radiograph plus at least two of the following criteria: (a) leukocytosis with > 12,000 WBC.mm^-3^ or leukopenia with < 4000 WBC.mm^-3^, (b) fever > 38.5°C or hypothermia < 36°C, and (c) increased secretions with purulent sputum and a positive bronchial aspirate | Presence of a new pulmonary infiltrate and/or progression of previous pulmonary infiltrates on a chest radiograph plus at least two of the following criteria: (a) leukocytosis with > 12,000 WBC.mm^-3^ or leukopenia with < 4000 WBC.mm^-3^, (b) fever > 38.5°C or hypothermia < 36°C, and (c) increased secretions with purulent sputum and a positive bronchial aspirate |
| Pleural effusion | Chest radiography with the presence of costophrenic angle blunting, displacement of adjacent anatomical structures, and blunting of the hemidiaphragmatic silhouette in the supine position | Chest radiography with the presence of costophrenic angle blunting, displacement of adjacent anatomical structures, and blunting of the hemidiaphragmatic silhouette in the supine position | Chest radiography with the presence of costophrenic angle blunting, displacement of adjacent anatomical structures, and blunting of the hemidiaphragmatic silhouette in the supine position |
| Atelectasis | Chest radiography with lung opacification with shift of the mediastinum, hilum, or hemidiaphragm towards the affected area, and compensatory overinflation in the adjacent non-atelectatic lung | Combination of SpO2 ≤ 96% during the air test and chest radiography with lung opacification with shift of the mediastinum, hilum, or hemidiaphragm towards the affected area, and compensatory overinflation in the adjacent non-atelectatic lung | Chest radiography with lung opacification with shift of the mediastinum, hilum, or hemidiaphragm towards the affected area, and compensatory overinflation in the adjacent non-atelectatic lung |
| Pneumothorax | Chest radiography with air in the pleural space with no vascular bed surrounding the visceral pleura | Chest radiography with air in the pleural space with no vascular bed surrounding the visceral pleura | Chest radiography with air in the pleural space with no vascular bed surrounding the visceral pleura |
| Bronchospasm | Presence of expiratory wheezing treated with bronchodilator | Presence of expiratory wheezing treated with bronchodilator | Presence of expiratory wheezing |
| ARDS: Acute Respiratory Distress Syndrome; SIRS: systemic inflammatory response syndrome; AECC: American–European consensus conference; WBC: White blood cells; PaO2: Arterial oxygen pressure: SpO2: peripheral oxygen saturation; FiO2: inspired oxygen fraction.  *Bernard GR, Artigas A, Brigham KL, et al. Report of the American-European consensus conference on ARDS: definitions, mechanisms, relevant outcomes and clinical trial coordination. The Consensus Committee. Intensive Care Med 1994;20:225–232.  **Ranieri VM, Rubenfeld GD, Thompson BT, et al. Acute respiratory distress syndrome: the Berlin Definition. JAMA 2012;307:2526–2533. | | | |

*Sensitivity analysis for patients with available plateau pressures*

**Table S2.** Patient characteristics (including only patients with available plateau pressures)

|  | Overall (N=1763) | CP <9 J.min^-1^ (N=906) | CP >9 J.min^-1^  (N=857) | SMD |
| --- | --- | --- | --- | --- |
| **Age (y)** | 55 ± 15 | 48 ± 13 | 62 ± 14 | 0.997 |
| **Sex (female)** | 994 (56%) | 621 (69%) | 373 (44%) | 0.521 |
| **Height (cm)** | 170 ± 9 | 170 ± 9 | 170 ± 9 | 0.040 |
| **Weight (kg)** | 100 ± 32 | 120 ± 23 | 84 ± 30 | 1.318 |
| **Body Mass Index (BMI)** | 37 ± 10 | 43 ± 7 | 31 ± 10 | 1.494 |
| **ASA Score** |  |  |  | 0.191 |
| 1 | 59 (3%) | 16 (2%) | 43 (5%) |  |
| 2 | 884 (50%) | 464 (51%) | 420 (49%) |  |
| 3 | 808 (46%) | 422 (47%) | 386 (45%) |  |
| 4 | 12 (1%) | 4 (0%) | 8 (1%) |  |
| **ARISCAT score** | 37 ± 9 | 39 ± 8 | 35 ± 10 | 0.392 |
| **Preoperative SPO_2_** | 96 ± 2 | 96 ± 2 | 97 ± 2 | 0.360 |
| **Respiratory infection** | 82 (5%) | 40 (4%) | 42 (5%) | 0.023 |
| **Preoperative anaemia** | 438 (25%) | 131 (14%) | 307 (36%) | 0.508 |
| **Heart failure** | 47 (3%) | 21 (2%) | 26 (3%) | 0.044 |
| **COPD** | 95 (5%) | 53 (6%) | 42 (5%) | 0.042 |
| **Active cancer** | 729 (41%) | 121 (13%) | 608 (71%) | 1.436 |
| **Preoperative haemoglobin (g.dL^-1^)** | 13 ± 3 | 14 ± 2 | 13 ± 4 | 0.151 |
| **Laparoscopic surgery** | 1128 (64%) | 700 (77%) | 428 (50%) | 0.592 |
| **Emergency procedure** | 17 (1%) | 15 (2%) | 2 (0%) | 0.147 |
| **Duration of surgery** | 210 ± 77 | 190 ± 68 | 220 ± 82 | 0.434 |
| **Specific procedure** |  |  |  | 1.465 |
| Bariatric | 680 (39%) | 565 (62%) | 115 (13%) |  |
| Bladder or Urologic | 76 (4%) | 24 (3%) | 52 (6%) |  |
| Bowel | 19 (1%) | 11 (1%) | 8 (1%) |  |
| Colorectal | 344 (20%) | 47 (5%) | 297 (35%) |  |
| Gastric | 86 (5%) | 16 (2%) | 70 (8%) |  |
| Gynaecology | 84 (5%) | 44 (5%) | 40 (5%) |  |
| Head Neck | 0 (0%) | 0 (0%) | 0 (0%) |  |
| Hepatic | 135 (8%) | 42 (5%) | 93 (11%) |  |
| Hernia | 31 (2%) | 23 (3%) | 8 (1%) |  |
| Kidney | 23 (1%) | 2 (0%) | 21 (2%) |  |
| Orthopaedic | 0 (0%) | 0 (0%) | 0 (0%) |  |
| Other | 193 (11%) | 117 (13%) | 76 (9%) |  |
| Other Gynaecology | 0 (0%) | 0 (0%) | 0 (0%) |  |
| Other Urologic | 0 (0%) | 0 (0%) | 0 (0%) |  |
| Pancreatic | 79 (4%) | 13 (1%) | 66 (8%) |  |
| Plastic | 0 (0%) | 0 (0%) | 0 (0%) |  |
| Vascular | 13 (1%) | 2 (0%) | 11 (1%) |  |
| **PPC** | 592 (34%) | 210 (23%) | 382 (45%) | 0.464 |
| *CP, chemical power; SMD, standardized mean difference; BMI, body mass index; ASA, American Society of Anesthesiologists; ARISCAT, Assess Respiratory Risk in Surgical Patients in Catalonia; SPO_2_, peripheral oxygen concentration; COPD, chronic obstructive pulmonary disease; PPC, postoperative pulmonary complications.* | | | | |

**Table S3.** Intraoperative ventilation parameters (including only patients with available plateau pressures)

|  | Overall (N=1763) | CP <9 J.min^-1^ (N=906) | CP >9 J.min^-1^  (N=857) | SMD |
| --- | --- | --- | --- | --- |
| **Tidal volume (mL)** | 440 ± 75 | 420 ± 68 | 470 ± 74 | 0.658 |
| **Tidal volume (mL.kgPBW^-1^)** | 7.5 ± 0.8 | 7.1 ± 0.4 | 7.8 ± 0.8 | 1.079 |
| **Respiratory rate (breaths.min^-1^)** | 16 ± 4 | 17 ± 3 | 14 ± 3 | 0.910 |
| **Plateau pressure (cmH_2_O)** | 22 ± 5 | 23 ± 4 | 21 ± 6 | 0.431 |
| **Peak pressure (cmH_2_O)** | 26 ± 6 | 27 ± 5 | 26 ± 6 | 0.312 |
| **PEEP (cmH_2_O)** | 8.0 ± 3.8 | 8.3 ± 4.0 | 7.7 ± 3.6 | 0.154 |
| **Static driving pressure (cmH_2_O)** | 14 ± 6 | 15 ± 5 | 13 ± 6 | 0.292 |
| **Dynamic driving pressure (cmH_2_O)** | 18 ± 6 | 19 ± 6 | 18 ± 6 | 0.312 |
| **FiO_2_ (%)** | 59 ± 18 | 43 ± 4 | 76 ± 9 | 4.505 |
| **Chemical power (J.min^-1^)** | 11 ± 4 | 8 ± 1 | 15 ± 2 | 4.505 |
| **Mechanical power (J.min^-1^)** | 13 ± 5 | 14 ± 5 | 12 ± 5 | 0.400 |
| *CP, chemical power; SMD, standardized mean difference; PBW, predicted bodyweight; PEEP, positive end-expiratory pressure; FiO_2_, fraction of inspiratory oxygen.* | | | | |

**Table S4.** Multivariable regression model to assess the associations of chemical and mechanical power with PPCs (including only patients with available plateau pressures)

| **Variables** | **OR** | **95% CI** | **p-value** |
| --- | --- | --- | --- |
| **Chemical power (J.min^-1^)** | 1.11 | 1.06, 1.15 | <0.001 |
| **Mechanical power (J.min^-1^)** | 1.03 | 1.00, 1.06 | 0.069 |
| **Age (y)** | 1.02 | 1.01, 1.03 | <0.001 |
| **Sex (female)** | 0.89 | 0.70, 1.13 | 0.4 |
| **Body Mass Index (BMI)** | 1.02 | 1.00, 1.04 | 0.014 |
| **ASA Score** |  |  |  |
| **1** | — | — |  |
| **2** | 1.75 | 0.89, 3.69 | 0.12 |
| **3** | 2.48 | 1.26, 5.29 | 0.012 |
| **4** | 2.59 | 0.61, 11.6 | 0.2 |
| **Preoperative SPO_2_** | 0.90 | 0.85, 0.95 | <0.001 |
| **Respiratory infection** | 1.39 | 0.84, 2.27 | 0.2 |
| **Preoperative anaemia** | 0.90 | 0.69, 1.17 | 0.5 |
| **Heart failure** | 1.64 | 0.88, 3.08 | 0.12 |
| **COPD** | 1.08 | 0.68, 1.70 | 0.8 |
| **Active cancer** | 1.00 | 0.72, 1.38 | >0.9 |
| **Laparoscopic surgery** | 0.62 | 0.47, 0.80 | <0.001 |
| **Emergency procedure** | 1.94 | 0.67, 5.54 | 0.2 |
| **PEEP (cmH_2_O)** | 0.96 | 0.94, 1.0 | 0.021 |
| **Duration of surgery (min)** | 1.00 | 1.00, 1.01 | <0.001 |
| *OR, odds ratio; 95%CI, 95% confidence interval; BMI, body mass index; ASA, American Society of Anesthesiologists; ARISCAT, Assess Respiratory Risk in Surgical Patients in Catalonia; SPO_2_, peripheral oxygen concentration; COPD, chronic obstructive pulmonary disease; PEEP, positive end-expiratory pressure.*  *Multivariable logistic regression was used to assess the associations of chemical and mechanical power with postoperative pulmonary complications (PPCs), adjusted for age, sex, BMI, ASA physical status, preoperative SPO_2_, respiratory infection, preoperative anaemia, heart failure, COPD, active cancer, laparoscopic vs. open abdominal surgery, emergency procedures, PEEP, and duration of surgery.* | | | |

*Sensitivity analysis for patients with presumably non-titrated FiO_2_*

**Table S5.** Patient characteristics (including only patients with FiO_2_ of 0.4, 0.5, or 0.8)

|  | Overall (N=1569) | CP <9 J.min^-1^ (N=944) | CP >9 J.min^-1^ (N=625) | SMD |
| --- | --- | --- | --- | --- |
| **Age (y)** | 58 ± 15 | 53 ± 16 | 65 ± 13 | 0.808 |
| **Sex (female)** | 778 (50%) | 538 (57%) | 240 (38%) | 0.379 |
| **Height (cm)** | 170 ± 9.2 | 170 ± 9.4 | 170 ± 8.9 | 0.155 |
| **Weight (kg)** | 90 ± 30 | 100 ± 31 | 72 ± 14 | 1.273 |
| **Body Mass Index (BMI)** | 32 ± 10 | 37 ± 10 | 26 ± 4.4 | 1.332 |
| **ASA Score** |  |  |  | 0.040 |
| 1 | 92 (6%) | 55 (6%) | 37 (6%) |  |
| 2 | 828 (53%) | 498 (53%) | 330 (53%) |  |
| 3 | 635 (40%) | 384 (41%) | 251 (40%) |  |
| 4 | 14 (1%) | 7 (1%) | 7 (1%) |  |
| **ARISCAT score** | 38 ± 9.6 | 40 ± 8.1 | 34 ± 11 | 0.639 |
| **Preoperative SPO_2_** | 97 ± 1.9 | 96 ± 1.8 | 97 ± 2.1 | 0.295 |
| **Respiratory infection** | 62 (4%) | 36 (4%) | 26 (4%) | 0.018 |
| **Preoperative anaemia** | 473 (30%) | 212 (22%) | 261 (42%) | 0.423 |
| **Heart failure** | 104 (7%) | 83 (9%) | 21 (3%) | 0.229 |
| **COPD** | 94 (6%) | 62 (7%) | 32 (5%) | 0.062 |
| **Active cancer** | 817 (52%) | 279 (30%) | 538 (86%) | 1.396 |
| **Preoperative haemoglobin (g.dL^-1^)** | 13 ± 5.5 | 14 ± 6.0 | 13 ± 4.6 | 0.148 |
| **Laparoscopic surgery** | 713 (45%) | 454 (48%) | 259 (41%) | 0.134 |
| **Emergency procedure** | 22 (1%) | 22 (2%) | 0 (0%) | 0.218 |
| **Duration of surgery** | 210 ± 80 | 200 ± 74 | 230 ± 84 | 0.352 |
| **Specific procedure** |  |  |  | 1.289 |
| Bariatric | 384 (24%) | 379 (40%) | 5 (1%) |  |
| Bladder or Urologic | 126 (8%) | 80 (8%) | 46 (7%) |  |
| Bowel | 14 (1%) | 7 (1%) | 7 (1%) |  |
| Colorectal | 431 (27%) | 163 (17%) | 268 (43%) |  |
| Gastric | 101 (6%) | 43 (5%) | 58 (9%) |  |
| Gynaecology | 52 (3%) | 32 (3%) | 20 (3%) |  |
| Head Neck | 0 (0%) | 0 (0%) | 0 (0%) |  |
| Hepatic | 130 (8%) | 52 (6%) | 78 (12%) |  |
| Hernia | 25 (2%) | 19 (2%) | 6 (1%) |  |
| Kidney | 25 (2%) | 6 (1%) | 19 (3%) |  |
| Orthopaedic | 0 (0%) | 0 (0%) | 0 (0%) |  |
| Other | 162 (10%) | 115 (12%) | 47 (8%) |  |
| Other Gynaecology | 0 (0%) | 0 (0%) | 0 (0%) |  |
| Other Urologic | 0 (0%) | 0 (0%) | 0 (0%) |  |
| Pancreatic | 100 (6%) | 39 (4%) | 61 (10%) |  |
| Plastic | 0 (0%) | 0 (0%) | 0 (0%) |  |
| Vascular | 19 (1%) | 9 (1%) | 10 (2%) |  |
| **PPC** | 507 (32%) | 230 (24%) | 277 (44%) | 0.430 |
| *CP, chemical power; SMD, standardized mean difference; BMI, body mass index; ASA, American Society of Anesthesiologists; ARISCAT, Assess Respiratory Risk in Surgical Patients in Catalonia; SPO_2_, peripheral oxygen concentration; COPD, chronic obstructive pulmonary disease; PPC, postoperative pulmonary complications.* | | | | |

**Table S6.** Intraoperative ventilation parameters (including only patients with FiO_2_ of 0.4, 0.5, or 0.8)

|  | Overall (N=1569) | CP <9 J.min^-1^ (N=944) | CP >9 J.min^-1^ (N=625) | SMD |
| --- | --- | --- | --- | --- |
| **Tidal volume (mL)** | 460 ± 77 | 450 ± 82 | 480 ± 67 | 0.339 |
| **Tidal volume (mL.kgPBW^-1^)** | 7.7 ± 0.8 | 7.5 ± 0.8 | 8.0 ± 0.8 | 0.690 |
| **Respiratory rate (breaths.min^-1^)** | 14 ± 4 | 15 ± 4 | 13 ± 2 | 0.508 |
| **Peak pressure (cmH_2_O)** | 25 ± 6 | 25 ± 6 | 25 ± 6 | 0.033 |
| **PEEP (cmH_2_O)** | 7.8 ± 4.2 | 7.7 ± 4.7 | 7.9 ± 3.4 | 0.054 |
| **Dynamic driving pressure (cmH_2_O)** | 17 ± 6 | 17 ± 6 | 17 ± 6 | 0.072 |
| **FiO_2_ (%)** | 58 ± 18 | 43 ± 5 | 80 ± 0 | 11.286 |
| **Chemical power (J.min^-1^)** | 11 ± 4 | 8 ± 1 | 16 ± 0 | 11.286 |
| **Mechanical power (J.min^-1^)** | 10 ± 4 | 11 ± 5 | 10 ± 3 | 0.175 |
| *CP, chemical power; SMD, standardized mean difference; PBW, predicted bodyweight; PEEP, positive end-expiratory pressure; FiO2, fraction of inspiratory oxygen.* | | | | |

**Table S7.** Multivariable regression model to assess the associations of chemical and mechanical power with PPCs (including only patients with FiO_2_ of 0.4, 0.5, or 0.8)

| **Variables** | **OR** | **95% CI** | **p-value** |
| --- | --- | --- | --- |
| **Chemical power (J.min^-1^)** | 1.09 | 1.06, 1.13 | <0.001 |
| **Mechanical power (J.min^-1^)** | 1.04 | 1.00, 1.09 | 0.061 |
| **Age (y)** | 1.02 | 1.01, 1.03 | <0.001 |
| **Sex (female)** | 0.93 | 0.73, 1.19 | 0.6 |
| **Body Mass Index (BMI)** | 1.01 | 0.99, 1.03 | 0.5 |
| **ASA Score** |  |  |  |
| **1** | — | — |  |
| **2** | 1.64 | 0.93, 3.04 | 0.10 |
| **3** | 2.13 | 1.19, 4.01 | 0.014 |
| **4** | 1.33 | 0.35, 4.96 | 0.7 |
| **Preoperative SPO_2_** | 0.90 | 0.84, 0.96 | 0.002 |
| **Respiratory infection** | 1.51 | 0.86, 2.64 | 0.15 |
| **Preoperative anaemia** | 1.03 | 0.79, 1.33 | 0.8 |
| **Heart failure** | 1.66 | 1.06, 2.60 | 0.027 |
| **COPD** | 1.11 | 0.69, 1.75 | 0.7 |
| **Active cancer** | 0.91 | 0.66, 1.25 | 0.5 |
| **Laparoscopic surgery** | 0.65 | 0.48, 0.88 | 0.005 |
| **Emergency procedure** | 1.39 | 0.50, 3.57 | 0.5 |
| **PEEP (cmH_2_O)** | 0.96 | 0.92, 0.99 | 0.008 |
| **Duration of surgery (min)** | 1.00 | 1.00, 1.01 | <0.001 |
| *OR, odds ratio; 95%CI, 95% confidence interval; BMI, body mass index; ASA, American Society of Anesthesiologists; ARISCAT, Assess Respiratory Risk in Surgical Patients in Catalonia; SPO_2_, peripheral oxygen concentration; COPD, chronic obstructive pulmonary disease; PEEP, positive end-expiratory pressure.*  *Multivariable logistic regression was used to assess the associations of chemical and mechanical power with postoperative pulmonary complications (PPCs), adjusted for age, sex, BMI, ASA physical status, preoperative SPO_2_, respiratory infection, preoperative anaemia, heart failure, COPD, active cancer, laparoscopic vs. open abdominal surgery, emergency procedures, PEEP, and duration of surgery.* | | | |

*Sensitivity analysis including all patients (missing data exclusions only)*

**Table S8.** Patient characteristics (including all patients)

|  | Overall (N=3084) | CP <9 J.min^-1^ (N=1932) | CP >9 J.min^-1^ (N=1152) | SMD |
| --- | --- | --- | --- | --- |
| **Age (y)** | 57 ± 16 | 54 ± 16 | 61 ± 14 | 0.493 |
| **Sex (female)** | 1663 (54%) | 1146 (59%) | 517 (45%) | 0.292 |
| **Height (cm)** | 170 ± 9.4 | 170 ± 9.4 | 170 ± 9.4 | 0.099 |
| **Weight (kg)** | 96 ± 32 | 100 ± 31 | 85 ± 31 | 0.533 |
| **Body Mass Index (BMI)** | 34 ± 11 | 37 ± 11 | 31 ± 10 | 0.552 |
| **ASA Score** |  |  |  | 0.115 |
| 1 | 178 (6%) | 119 (6%) | 59 (5%) |  |
| 2 | 1577 (51%) | 1018 (53%) | 559 (49%) |  |
| 3 | 1302 (42%) | 780 (40%) | 522 (45%) |  |
| 4 | 26 (1%) | 14 (1%) | 12 (1%) |  |
| 5 | 1 (0%) | 1 (0%) | 0 (0%) |  |
| **ARISCAT score** | 38 ± 9.6 | 39 ± 8.1 | 35 ± 11 | 0.500 |
| **Preoperative SPO_2_** | 97 ± 2.0 | 97 ± 1.9 | 97 ± 2.1 | 0.105 |
| **Respiratory infection** | 146 (5%) | 94 (5%) | 52 (5%) | 0.017 |
| **Preoperative anaemia** | 803 (26%) | 408 (21%) | 395 (34%) | 0.298 |
| **Heart failure** | 253 (8%) | 190 (10%) | 63 (5%) | 0.165 |
| **COPD** | 185 (6%) | 126 (7%) | 59 (5%) | 0.060 |
| **Active cancer** | 1347 (44%) | 590 (31%) | 757 (66%) | 0.752 |
| **Preoperative haemoglobin (g.dL^-1^)** | 13 ± 4.7 | 14 ± 4.4 | 13 ± 5.0 | 0.066 |
| **Laparoscopic surgery** | 1489 (48%) | 942 (49%) | 547 (47%) | 0.026 |
| **Emergency procedure** | 43 (1%) | 39 (2%) | 4 (0%) | 0.155 |
| **Duration of surgery** | 190 ± 90 | 190 ± 89 | 200 ± 90 | 0.192 |
| **Specific procedure** |  |  |  | 0.649 |
| Bariatric | 911 (30%) | 738 (38%) | 173 (15%) |  |
| Bladder or Urologic | 191 (6%) | 129 (7%) | 62 (5%) |  |
| Bowel | 44 (1%) | 29 (2%) | 15 (1%) |  |
| Colorectal | 683 (22%) | 300 (16%) | 383 (33%) |  |
| Gastric | 184 (6%) | 95 (5%) | 89 (8%) |  |
| Gynecology | 145 (5%) | 93 (5%) | 52 (5%) |  |
| Head Neck | 0 (0%) | 0 (0%) | 0 (0%) |  |
| Hepatic | 262 (8%) | 144 (7%) | 118 (10%) |  |
| Hernia | 57 (2%) | 41 (2%) | 16 (1%) |  |
| Kidney | 47 (2%) | 21 (1%) | 26 (2%) |  |
| Orthopaedic | 0 (0%) | 0 (0%) | 0 (0%) |  |
| Other | 313 (10%) | 209 (11%) | 104 (9%) |  |
| Other Gynaecology | 0 (0%) | 0 (0%) | 0 (0%) |  |
| Other Urologic | 0 (0%) | 0 (0%) | 0 (0%) |  |
| Pancreatic | 199 (6%) | 105 (5%) | 94 (8%) |  |
| Plastic | 0 (0%) | 0 (0%) | 0 (0%) |  |
| Vascular | 48 (2%) | 28 (1%) | 20 (2%) |  |
| **PPC** | 983 (32%) | 493 (26%) | 490 (43%) | 0.365 |
| *CP, chemical power; SMD, standardized mean difference; BMI, body mass index; ASA, American Society of Anesthesiologists; ARISCAT, Assess Respiratory Risk in Surgical Patients in Catalonia; SPO_2_, peripheral oxygen concentration; COPD, chronic obstructive pulmonary disease; PPC, postoperative pulmonary complications.* | | | | |

**Table S9.** Intraoperative ventilation parameters (including all patients)

|  | Overall (N=3084) | CP <9 J.min^-1^ (N=1932) | CP >9 J.min^-1^ (N=1152) | SMD |
| --- | --- | --- | --- | --- |
| **Tidal volume (mL)** | 460 ± 89 | 460 ± 93 | 470 ± 81 | 0.190 |
| **Tidal volume (mL.kgPBW^-1^)** | 7.7 ± 1.0 | 7.5 ± 1.1 | 7.9 ± 1.0 | 0.326 |
| **Respiratory rate (breaths.min^-1^)** | 14 ± 4 | 15 ± 4 | 14 ± 3 | 0.205 |
| **Peak pressure (cmH_2_O)** | 25 ± 6 | 24 ± 6 | 25 ± 7 | 0.129 |
| **Dynamic driving pressure (cmH_2_O)** | 17 ± 6 | 17 ± 6 | 18 ± 6 | 0.143 |
| **PEEP (cmH_2_O)** | 7.6 ± 4.4 | 7.6 ± 4.7 | 7.5 ± 3.8 | 0.015 |
| **FiO_2_ (%)** | 54 ± 17 | 43 ± 5 | 74 ± 11 | 3.722 |
| **Chemical Power (J.min^-1^)** | 10 ± 4 | 7.5 ± 1 | 15 ± 3 | 3.722 |
| **Mechanical Power (J.min^-1^)** | 11 ± 5 | 11 ± 5 | 11 ± 4 | 0.006 |
| *CP, chemical power; SMD, standardized mean difference; PBW, predicted bodyweight; PEEP, positive end-expiratory pressure; FiO_2_, fraction of inspiratory oxygen.* | | | | |

**Table S10.** Multivariable regression model to assess the associations of chemical and mechanical power with PPCs (including all patients)

| **Variables** | **OR** | **95% CI** | **p-value** |
| --- | --- | --- | --- |
| **Chemical power (J.min^-1^)** | 1.08 | 1.05, 1.11 | <0.001 |
| **Mechanical power (J.min^-1^)** | 1.04 | 1.01, 1.06 | 0.005 |
| **Age (y)** | 1.02 | 1.01, 1.03 | <0.001 |
| **Sex (female)** | 0.95 | 0.80, 1.14 | 0.6 |
| **Body Mass Index (BMI)** | 1.01 | 0.99, 1.02 | 0.4 |
| **ASA Score** |  |  |  |
| **1** | — | — |  |
| **2** | 1.85 | 1.21, 2.92 | 0.006 |
| **3** | 2.69 | 1.74, 4.30 | <0.001 |
| **4** | 2.29 | 0.89, 5.92 | 0.085 |
| **5** | 0.00 |  | >0.9 |
| **Preoperative SPO_2_** | 0.91 | 0.87, 0.96 | <0.001 |
| **Respiratory infection** | 1.50 | 1.02, 2.19 | 0.035 |
| **Preoperative anaemia** | 1.07 | 0.88, 1.30 | 0.5 |
| **Heart failure** | 1.39 | 1.03, 1.86 | 0.030 |
| **COPD** | 1.18 | 0.84, 1.64 | 0.3 |
| **Active cancer** | 0.98 | 0.79, 1.22 | 0.9 |
| **Laparoscopic surgery** | 0.63 | 0.50, 0.78 | <0.001 |
| **Emergency procedure** | 1.45 | 0.72, 2.82 | 0.3 |
| **PEEP (cmH_2_O)** | 0.97 | 0.95, 0.99 | 0.005 |
| **Duration of surgery (min)** | 1.01 | 1.00, 1.01 | <0.001 |
| *OR, odds ratio; 95%CI, 95% confidence interval; BMI, body mass index; ASA, American Society of Anesthesiologists; ARISCAT, Assess Respiratory Risk in Surgical Patients in Catalonia; SPO2, peripheral oxygen concentration; COPD, chronic obstructive pulmonary disease; PEEP, positive end-expiratory pressure.*  *Multivariable logistic regression was used to assess the associations of chemical and mechanical power with postoperative pulmonary complications (PPCs), adjusted for age, sex, BMI, ASA physical status, preoperative SPO2, respiratory infection, preoperative anaemia, heart failure, COPD, active cancer, laparoscopic vs. open abdominal surgery, emergency procedures, PEEP, and duration of surgery.* | | | |

*Sensitivity analysis – adjustment of the primary analysis for trial effects*

**Table S11.** Multivariable regression model to assess the associations of chemical and mechanical power with PPCs (adjustment of the primary analysis for trial effects)

| **Variables** | **OR** | **95% CI** | **p-value** |
| --- | --- | --- | --- |
| **Chemical power (J.min^-1^)** | 1.08 | 1.03, 1.14 | 0.001 |
| **Mechanical power (J.min^-1^)** | 1.05 | 1.02, 1.09 | 0.002 |
| **Age (y)** | 1.02 | 1.01, 1.03 | <0.001 |
| **Sex (female)** | 0.96 | 0.79, 1.17 | 0.7 |
| **Body Mass Index (BMI)** | 1.01 | 1.0, 1.03 | 0.2 |
| **ASA Score** |  |  |  |
| **1** | — | — |  |
| **2** | 2.01 | 1.26, 3.30 | 0.004 |
| **3** | 2.98 | 1.85, 4.96 | <0.001 |
| **4** | 2.32 | 0.85, 6.31 | 0.10 |
| **Preoperative SPO_2_** | 0.92 | 0.87, 0.96 | <0.001 |
| **Respiratory infection** | 1.53 | 1.00, 2.31 | 0.046 |
| **Preoperative anaemia** | 1.06 | 0.86, 1.31 | 0.6 |
| **Heart failure** | 1.29 | 0.92, 1.79 | 0.14 |
| **COPD** | 1.12 | 0.79, 1.60 | 0.5 |
| **Active cancer** | 0.91 | 0.71, 1.15 | 0.4 |
| **Laparoscopic surgery** | 0.59 | 0.46, 0.76 | <0.001 |
| **Emergency procedure** | 1.93 | 0.92, 4.04 | 0.080 |
| **PEEP (cmH_2_O)** | 0.97 | 0.94, 0.99 | 0.009 |
| **Duration of surgery** | 1.01 | 1.00, 1.01 | <0.001 |
| **Trial** |  |  |  |
| **A** | — | — |  |
| **B** | 0.75 | 0.50, 1.11 | 0.15 |
| **C** | 0.85 | 0.53, 1.37 | 0.5 |
| *OR, odds ratio; 95%CI, 95% confidence interval; BMI, body mass index; ASA, American Society of Anesthesiologists; ARISCAT, Assess Respiratory Risk in Surgical Patients in Catalonia; SPO2, peripheral oxygen concentration; COPD, chronic obstructive pulmonary disease; PEEP, positive end-expiratory pressure.*  *Multivariable logistic regression was used to assess the associations of chemical and mechanical power with postoperative pulmonary complications (PPCs), adjusted for age, sex, BMI, ASA physical status, preoperative SPO2, respiratory infection, preoperative anaemia, heart failure, COPD, active cancer, laparoscopic vs. open abdominal surgery, emergency procedures, PEEP, duration of surgery, and different trial participation.* | | | |
