# Supplementary material for: Individual and combined effects of chemical and mechanical power on postoperative pulmonary complications: a secondary analysis of the REPEAT study
Source: Anaesthesia. 2025 Aug 19;80(12):1510–8. doi: 10.1111/anae.16725 (PMC12614417; doi:10.1111/anae.16725)
Supplement: Supplementary file 3 — Figure S1. Chemical power and the corresponding FIO2 in patients with and without postoperative pulmonary complications. Figure S2. Probability of postoperative pulmonary complications over the ranges of chemical and mechanical power (including only patients with available plateau pressures). Figure S3. Probability of postoperative pulmonary complications associated with chemical and mechanical power (including only patients with available plateau pressures). Figure S4. Probability of postoperative pulmonary complications over the ranges of chemical and mechanical power (including only patients with FIO2 of 0.4, 0.5 or 0.8). Figure S5. Probability of postoperative pulmonary complications associated with chemical and mechanical power (including only patients with FIO2 of 0.4, 0.5 or 0.8). Figure S6. Probability of postoperative pulmonary complications over the ranges of chemical and mechanical power (including all patients). Figure S7. Probability of postoperative pulmonary complications associated with chemical and mechanical power (including all patients). [file ANAE-80-1510-s003.docx]

***Appendix S3: Supplemental Figures***


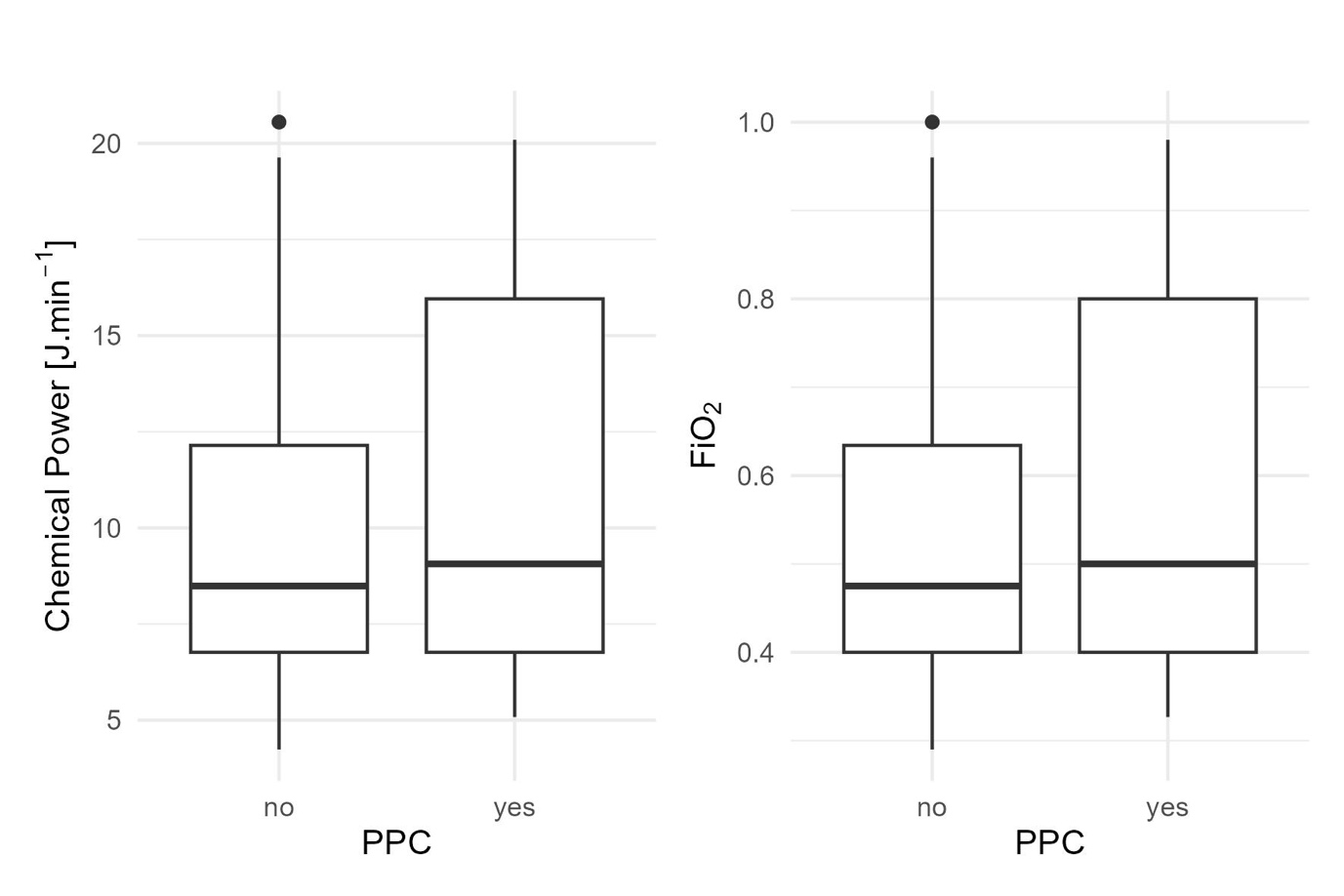


**Figure S1.** Chemical power and the corresponding fraction of inspiratory oxygen (FiO_2_) in patients with and without postoperative pulmonary complication (PPC)

*Sensitivity analysis for patients with available plateau pressures*

**
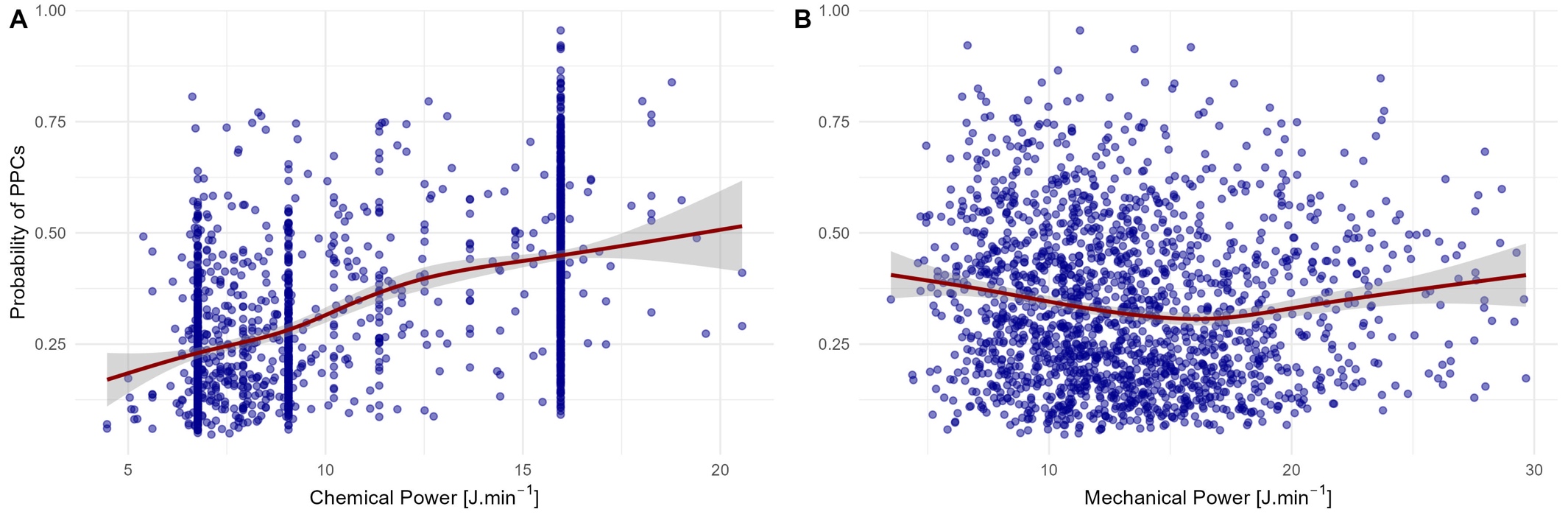
Figure S2.** Probability of postoperative pulmonary complications (PPCs) over the ranges of chemical (A) and mechanical (B) power (including only patients with available plateau pressures)


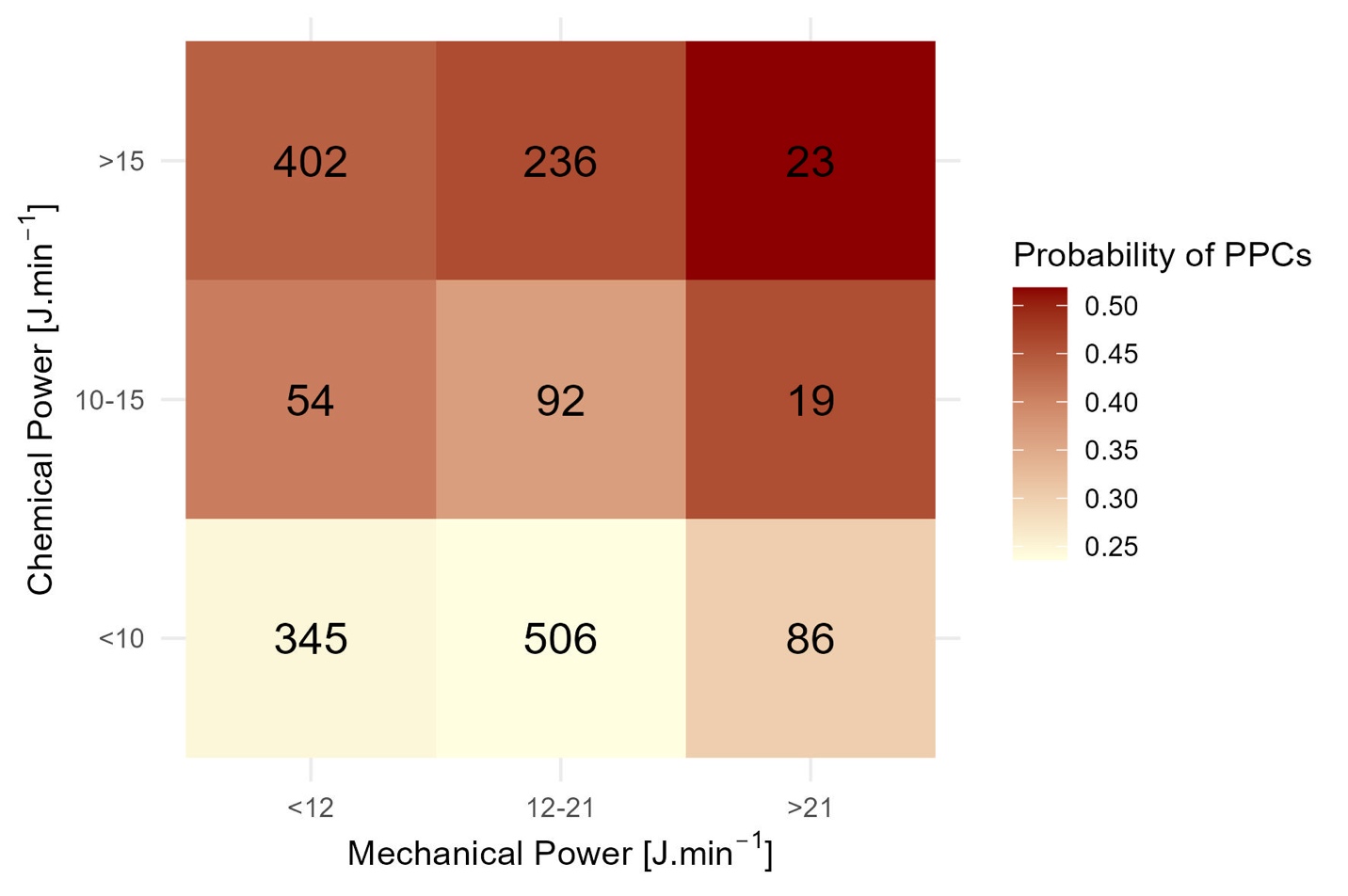


**Figure S3.** Probability of postoperative pulmonary complications (PPCs) associated with chemical and mechanical power (including only patients with available plateau pressures)

*Sensitivity analysis for patients with presumably non-titrated FiO_2_*


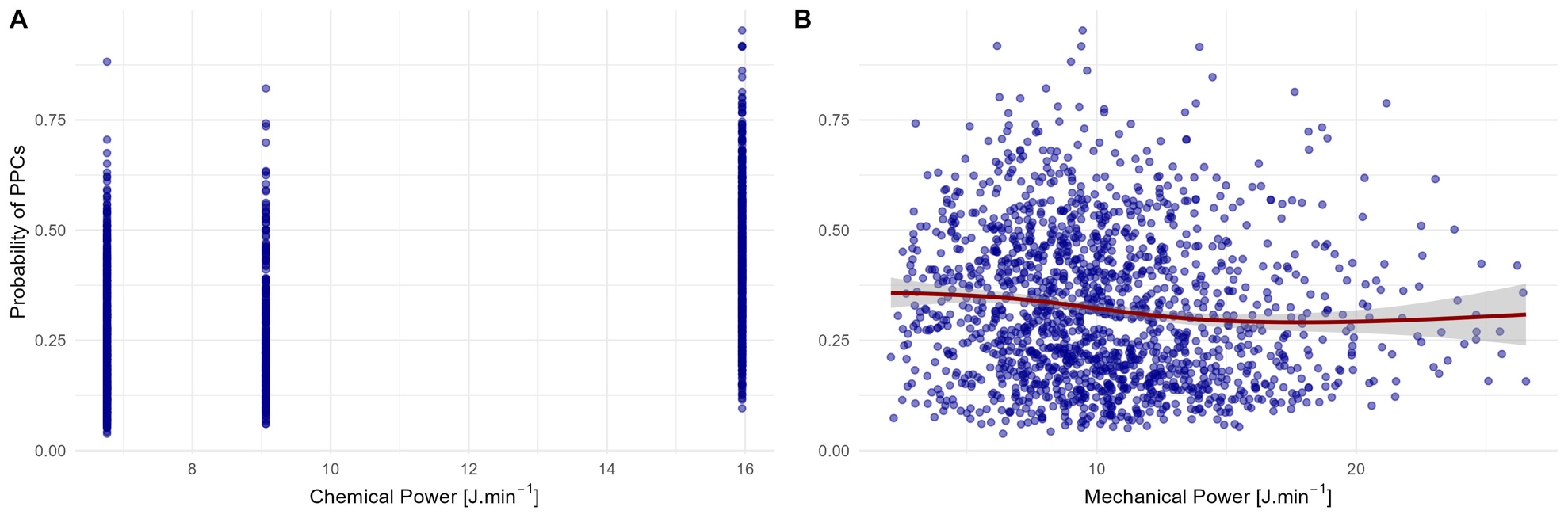


**Figure S4.** Probability of postoperative pulmonary complications (PPCs) over the ranges of chemical (A) and mechanical (B) power (including only patients with FiO_2_ of 0.4, 0.5, or 0.8)


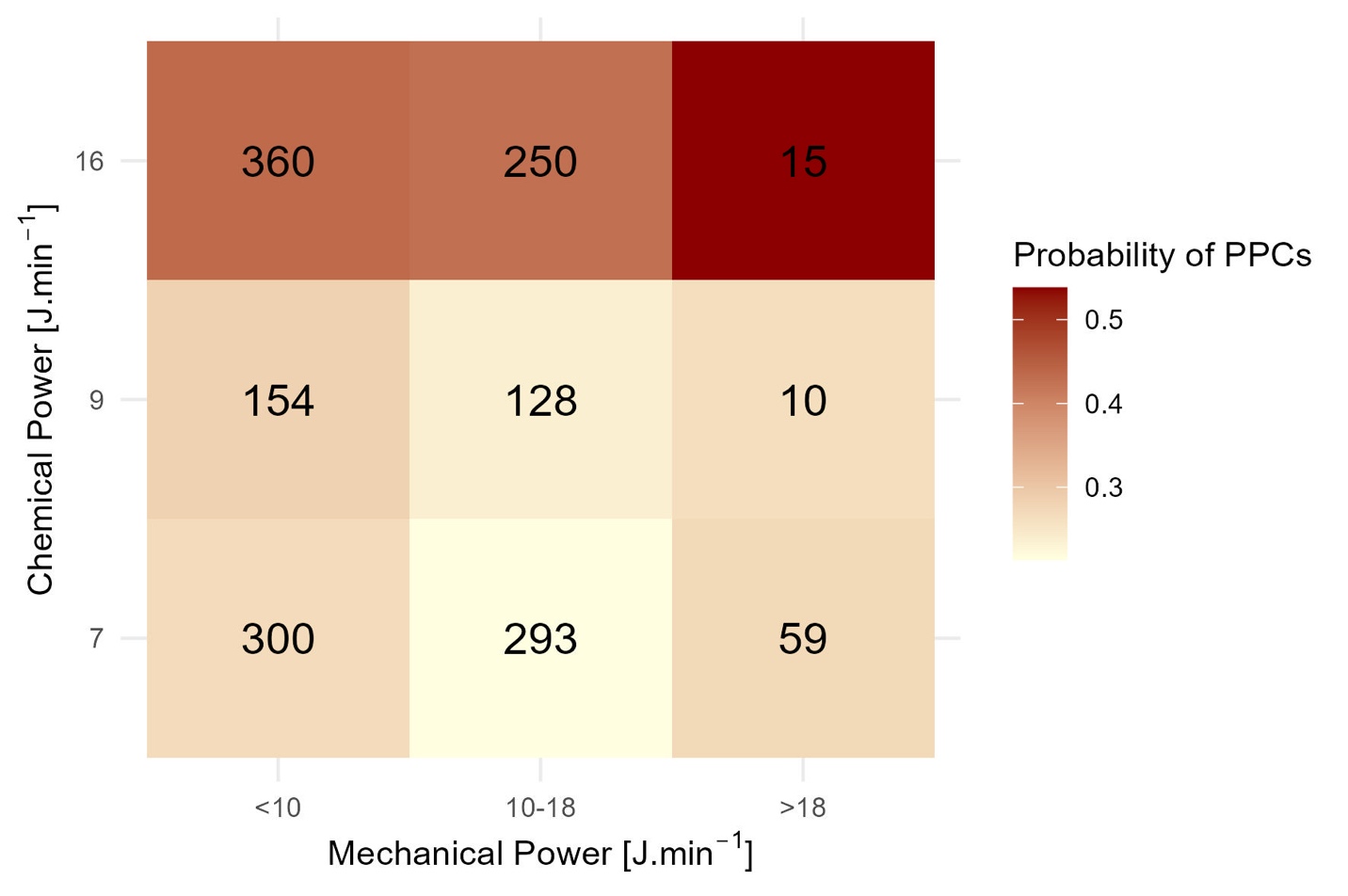


**Figure S5.** Probability of postoperative pulmonary complications (PPCs) associated with chemical and mechanical power (including only patients with FiO_2_ of 0.4, 0.5, or 0.8)

*Sensitivity analysis including all patients (missing data exclusions only)*


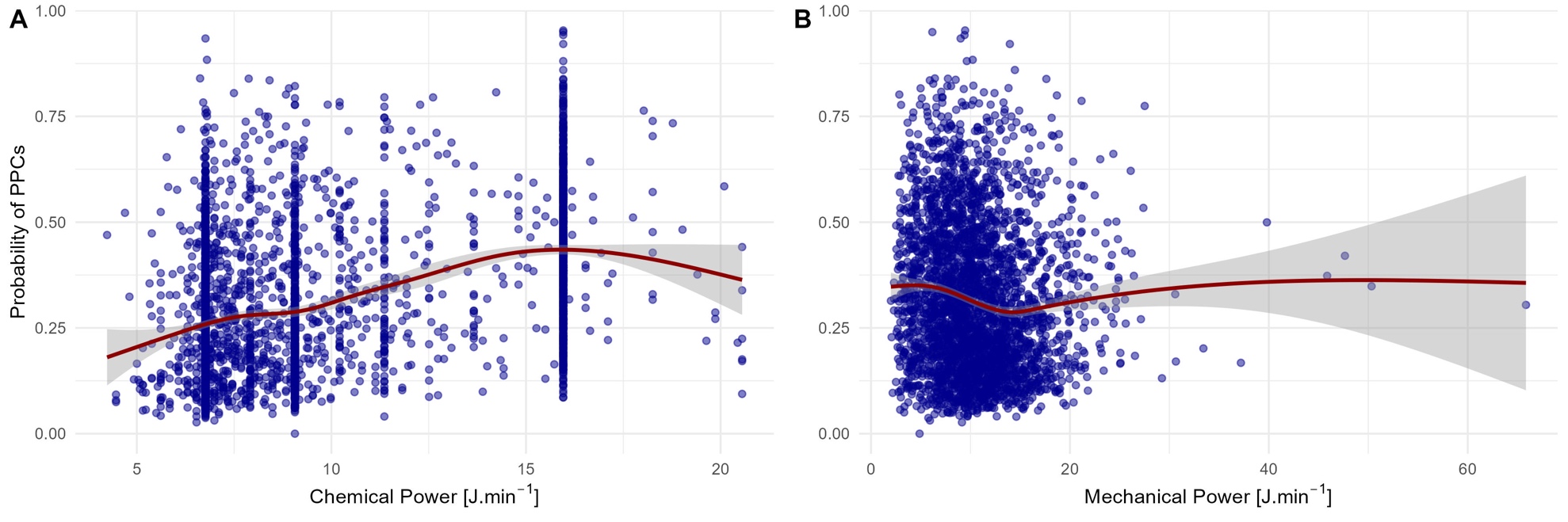


**Figure S6.** Probability of postoperative pulmonary complications (PPCs) over the ranges of chemical (A) and mechanical (B) power (including all patients)


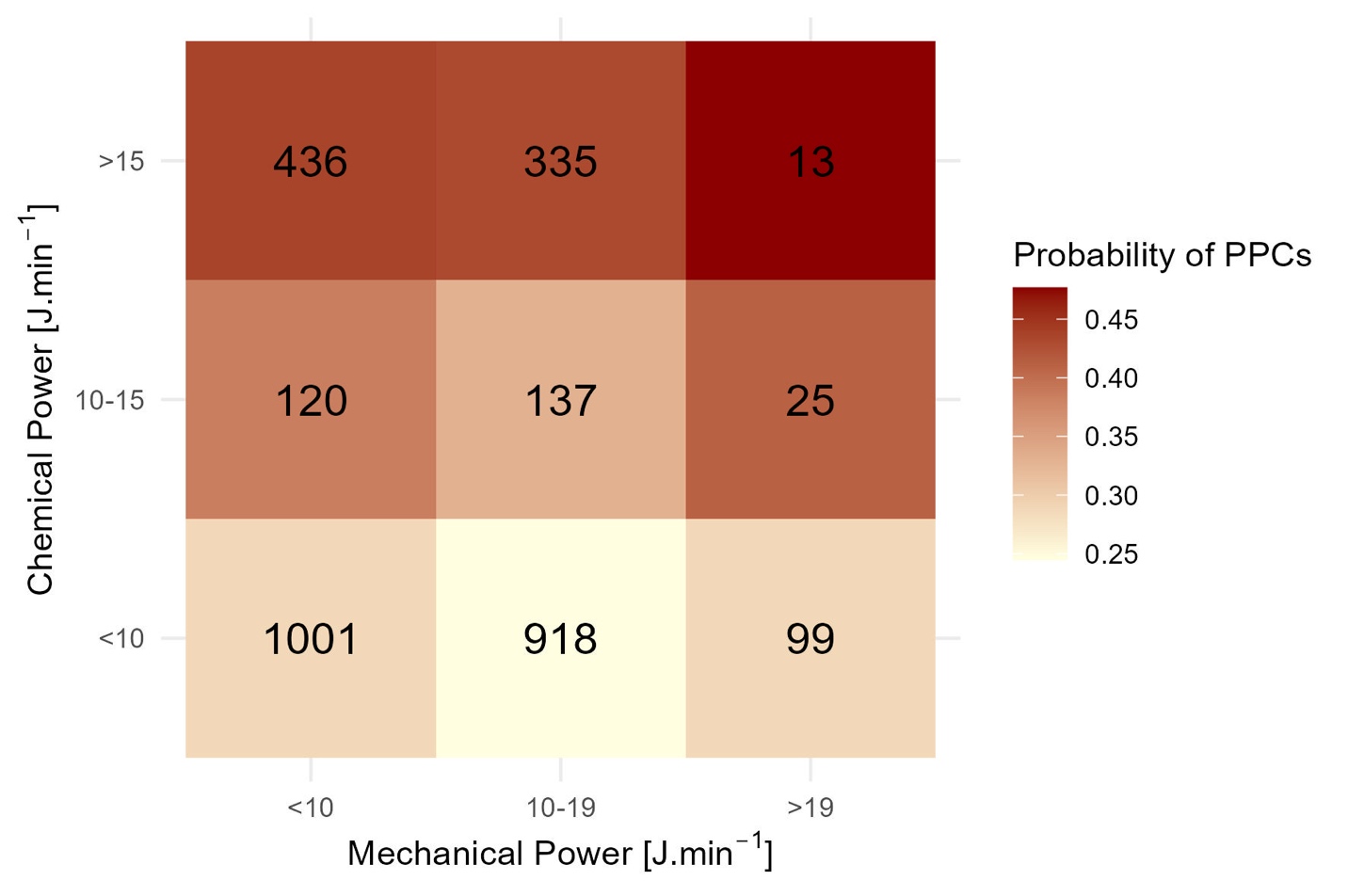


**Figure S7.** Probability of postoperative pulmonary complications (PPCs) associated with chemical and mechanical power (including all patients)
