# Supplementary material for: Individual and combined effects of chemical and mechanical power on postoperative pulmonary complications: a secondary analysis of the REPEAT study
Source: Anaesthesia. 2025 Aug 19;80(12):1510–8. doi: 10.1111/anae.16725 (PMC12614417; doi:10.1111/anae.16725)
Supplement: Supplementary file 4 — Plain Language Summary. [file ANAE-80-1510-s001.docx]

**Plain Language Summary**

When people have surgery, they often need extra oxygen and a machine to help them breathe. But this can sometimes put stress on the lungs and cause problems afterward. In this study, doctors wanted to see if the amount of energy used by the oxygen and the breathing machine (called 'chemical power' and 'mechanical power') could be linked to lung problems after surgery. Doctors looked at data from three big studies involving patients who had surgery with breathing support. They measured how much chemical and mechanical power was used during surgery and averaged it over time. Then, they checked to see if people who had more of this power used during surgery were more likely to have breathing or lung problems afterward. They also checked if using both types of power together made things worse. Out of 3837 patients, 2492 had complete information and were included in the study. On average, the chemical power used was about 10.2 and the mechanical power was about 10.5 (measured in special units called Joules per minute). For every small increase in chemical power, the chance of lung problems after surgery went up by 8%. For the same increase in mechanical power, the chance went up by 5%. The two types of power didn’t seem to make each other worse when used together. The study showed that both chemical and mechanical power during surgery can raise the risk of lung problems afterward. More research is needed to understand if one causes the other, or if something else is going on.
